# Glycans on non-structural protein 1 prevent premature T-cell mediated dengue virus clearance

**DOI:** 10.1038/s44321-025-00311-6

**Published:** 2025-09-17

**Authors:** Fakhriedzwan Idris, Justin Seng Geap Ooi, Donald Heng Rong Ting, Eunice Tze Xin Tan, Corrine Wan, Peter I Benke, Jan K Marzinek, Jack M Copping, Qin Hui Li, Lu Yi Ng, Sheau Yng Lim, Ian Walsh, Jane R Allison, Peter J Bond, Federico Torta, Terry Nguyen-Khuong, Kuan Rong Chan, Sylvie Alonso

**Affiliations:** 1 Infectious Diseases Translational Research Programme, Department of Microbiology and Immunology, Yong Loo Lin School of Medicine, National University of Singapore, Singapore, 117545 Singapore; 2 Immunology Programme, Life Sciences Institute, National University of Singapore, Singapore, 117456 Singapore; 3 Emerging Infectious Diseases Programme, Duke-NUS Medical School, Singapore, 169857 Singapore; 4 Bioprocessing Technology Institute (BTI), Agency for Science, Technology and Research (A*STAR), Singapore, 138668 Singapore; 5 Precision Medicine Translational Research Programme and Department of Biochemistry, Yong Loo Lin School of Medicine, National University of Singapore, Singapore, 117596 Singapore; 6 Singapore Lipidomics Incubator, Life Sciences Institute, National University of Singapore, Singapore, 117456 Singapore; 7 Bioinformatics Institute (BII), Agency for Science, Technology and Research (A*STAR), Singapore, 138671 Singapore; 8 Biomolecular Interaction Centre, Digital Life Institute, Maurice Wilkins Centre for Molecular Biodiscovery, University of Auckland, Private Bag, Auckland, 92019 New Zealand; 9 School of Biological Sciences, University of Auckland, Auckland, 1010 New Zealand; 10 Immunology Translational Research Programme, Department of Microbiology and Immunology, Yong Loo Lin School of Medicine, National University of Singapore, Singapore, 117456 Singapore; 11 Department of Biological Sciences, National University of Singapore, Singapore, 117558 Singapore; 12 Signature Research Program in Cardiovascular and Metabolic Disorders, Duke-National University of Singapore (NUS) Medical School, Singapore, 169857 Singapore; 13 National Centre for Infectious Diseases, Singapore, 308442 Singapore

**Keywords:** Dengue, Glycosylation, PD-1, NS1, T-cell Apoptosis, Immunology, Microbiology, Virology & Host Pathogen Interaction

## Abstract

Non-structural protein 1 (NS1) of dengue virus (DENV) harbours two conserved N-glycosylation sites at positions 130 and 207, whose biological roles have remained elusive. Using a clinically relevant mouse model of severe dengue, we showed that DENV that lacked N207 glycans on NS1 was significantly attenuated, and this phenotype was dominant over wild-type virulent DENV. Mice infected with this mutant exhibited accelerated viral clearance, milder lymphopenia and more functional DENV-specific CD8^+^ T cells. Bulk and single-cell RNA sequencing, cytokine measurements and immune-phenotyping revealed blunted innate inflammatory responses early post-infection, which correlated with reduced PD-L1 expression on innate immune cells and reduced PD-1^+^ T-cells in mice infected with de-glycosylated DENV. PD-1 blockade demonstrated the involvement of premature T-cell apoptosis through the PD-L1/PD-1 axis in DENV pathogenesis. Collectively, our findings support that N207-de-glycosylated NS1 inhibits early inflammatory responses, which restricts PD-L1 upregulation on innate immune cells, which in turn limits PD-L1/PD-1 mediated T-cell apoptosis. Our study uncovers a novel immune evasion strategy and identifies PD-L1/PD-1 as a novel mechanism of dengue immunopathogenesis.

## Synopsis

This study reports a novel immune evasion strategy evolved by dengue virus (DENV) to dampen T-cell mediated viral clearance. The mechanism involves highly conserved N-linked glycans at position 207 on non-structural protein (NS1).

- NS1 lacking N207-glycans suppresses the early pro-inflammatory response during DENV infection, which dampens PD-L1 expression on innate immune cells, thereby limiting interactions with PD-1 on T-cells.
- DENV lacking N207-linked glycans on NS1 is attenuated in mice with improved T-cell mediated viral clearance.
- PD-1 blockade protected mice infected with WT DENV.
- The PD-L1/PD-1 axis is a novel pathway involved in dengue immunopathogenesis.

## Abstract (web summary)

This study reports a novel immune evasion strategy evolved by dengue virus (DENV) to dampen T-cell mediated viral clearance. The mechanism involves highly conserved N-linked glycans at position 207 on non-structural protein (NS1).

## Graphical abstract

**Figure:**
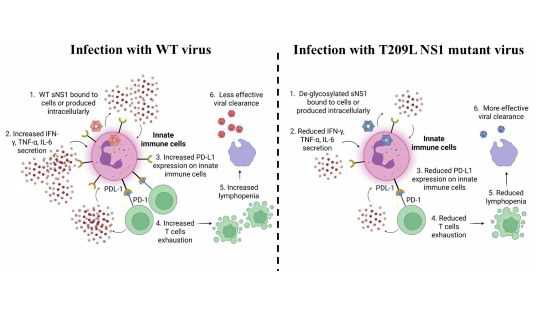

**Figure:**
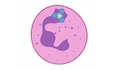

The paper explained **Problem**

Identified as one of the top ten global health threats by the WHO in 2025, dengue affects millions of individuals every year in more than 130 tropical and subtropical countries. The lack of effective antivirals and vaccines can be partially attributed to the complexity of dengue pathogenesis and the heterogeneity among the dengue virus (DENV) strains, which have adapted to different geographical areas. Non-structural protein 1 (NS1) is secreted by DENV-infected cells and exerts diverse biological activities that contribute to dengue pathogenesis. NS1 contains two highly conserved N-glycosylation sites at positions 130 and 207, which are completely conserved across all the DENV strains and the entire orthoflavivirus genus, suggesting important biological function(s). However, the role(s) of these N-linked glycans has not been studied comprehensively and remain largely unknown.

**Results**

Using a clinically relevant mouse model of severe dengue, this study reports the role of N207-linked glycans on NS1 in DENV pathogenesis. Using a N207-de-glycosylated DENV mutant and through a multidimensional approach, the findings support that N207-de-glycosylated NS1 inhibits early inflammatory responses, thereby limiting PD-L1 upregulation on innate immune cells and restricting PD-L1/PD-1-mediated T-cell apoptosis, ultimately improving killing of DENV-infected cells.

**Impact**

The study uncovers a novel immune evasion strategy employed by DENV that involves N207-linked glycans on NS1 and identifies the PD-L1/PD-1 axis as a previously unrecognized mechanism of dengue immunopathogenesis, which may inform future therapeutic strategies. Understanding the functional significance of highly conserved N-linked glycosylation sites on pathogens is critical and can lead to novel treatment options.

## Introduction

Dengue is a mosquito-borne viral disease that poses a significant threat in the tropical and subtropical regions, impacting approximately 390 million individuals across more than 125 countries (Bhatt et al, 2013). Projections indicate that by 2080, this number could rise to 2.25 billion people (Messina et al, 2019). In these endemic countries, dengue is a major public health concern due to the limited efficacy of existing dengue vaccines, and the lack of specific antiviral treatment (Idris et al, 2021). Symptomatic dengue causes flu-like symptoms with fever and rash. However, it can develop into severe illness known as dengue haemorrhagic fever (DHF), characterised by coagulopathy, vascular leakage, and thrombocytopenia, which may further progress into dengue shock syndrome (DSS) (Martina et al, 2009). The infection is caused by dengue virus (DENV) that belongs to the Flaviviridae family, Orthoflavivirus genus. DENV strains have been classified under four antigenically distinct serotypes (DENV1-4) that co-circulate with unpredictable dominance patterns. Like other flaviviruses, DENV genome is a positive-sense, single stranded RNA of approximately 10.7 kb in size and encodes three structural and seven non-structural (NS) proteins. Among which, NS1 is highly conserved across DENV1-4 and comprises of 352 amino acids with a molecular weight ranging from 40 to 50 kDa, depending on its glycosylation status (Flamand et al, 1999). NS1 monomers and dimers are found intracellularly, with the dimers often membrane-associated, while the secreted, soluble form of NS1 (sNS1) has been described as an atypical barrel-shaped hexamer with a central lipid cargo (Gutsche et al, 2011). A recent study however has challenged this model and reported that sNS1 exists predominantly in either ‘loose’ or ‘stable’ tetrameric form with differential susceptibility to antibodies (Shu et al, 2022).

While the main role of intracellular NS1 is to support DENV genome replication (Zhang et al, 2023), in vitro and in vivo studies have reported a variety of biological activities for secreted sNS1, including interfering with complement activation pathways (Lee et al, 2022) and inducing endothelial hyperpermeability. The latter involved shedding of the glycocalyx lining the intravascular endothelial layer (Biering et al, 2021; Puerta-Guardo et al, 2019) and/or Toll-like receptor 4 (TLR4)-mediated production of pro-inflammatory cytokines (Modhiran et al, 2015). Other studies have also shown that sNS1 is highly immunogenic (Freire et al, 2017; Stettler et al, 2016), and that anti-NS1 antibodies afforded protection against severe disease in animal models (Bailey et al, 2019; Beatty et al, 2015; Gonçalves et al, 2015; Richner et al, 2017).

DENV NS1 has two N-linked glycans at N130 and N207 position, which are conserved across the four DENV serotypes, and whose sugar composition is site-specific and depends on the cell type in which NS1 protein is produced (Thiemmeca et al, 2016). DENV NS1 produced in mammalian cells typically displays complex glycans at N130, whereas N207 sugars mainly consist of high-mannose (Pryor and Wright, 1994). These N-glycans have been proposed to confer stability to the NS1 dimer and to be involved in the secretion of multimeric sNS1 (Somnuke et al, 2011; Thiemmeca et al, 2016). Moreover, abrogation of N130 or both N-glycosylation sites on NS1 resulted in reduced virus growth in vitro (Crabtree et al, 2005; Pryor and Wright, 1994; Tajima et al, 2008). On the other hand, N207 glycans were found to play an essential role in sNS1-induced glycocalyx layer disruption in endothelial cell cultures (Wang et al, 2019). In vivo studies reported that lack of glycans on NS1 at either site led to significant attenuation of DENV neurovirulence in suckling mice (Crabtree et al, 2005; Fang et al, 2023). The authors speculated that lower sNS1 levels in circulation were responsible for the observed attenuated phenotype, although no experimental evidence was provided to support this hypothesis.

Here, we investigated the role of N-linked glycans on NS1 produced by a DENV2 strain from the Cosmopolitan genotype. DENV2 Cosmopolitan is the most widespread DENV2 genotype that has been circulating in Asia-Pacific, Middle East, and Africa for decades and has caused recurrent outbreaks associated with severe dengue (Yenamandra et al, 2021). More recently, Cosmopolitan DENV2 has expanded further into other parts of the world like South America (Gräf et al, 2023), underscoring the exceptional fitness of these strains.

Using a non-mouse adapted DENV2 Cosmopolitan strain (namely D2Y98P), our lab has established a clinically relevant mouse model of severe dengue whereby the disease kinetic observed in the infected mice closely mirrors the kinetic seen in dengue patients who experience severe clinical manifestations after the peak of viremia, during the defervescence stage (Tan et al, 2010). The infected mice develop progressive vascular leakage, as well as liver and intestine damage, which are accompanied by elevated pro-inflammatory cytokines, thrombocytopenia and lymphopenia (Tan et al, 2010). Leveraging this mouse model, we hereby report a novel immune evasion strategy mediated by N207 glycans on NS1 and a novel mechanism of dengue immune pathogenesis.

## Results

### Generation and characterization of de-glycosylated NS1 DENV mutants

DENV mutants that lack the glycan motifs on either N130 or N207 sites were generated by substituting one of the amino acids in the glycosylation motif N-X-S/T (X represents any amino acid except Proline). All six mutants were viable and displayed plaque morphology that was comparable to parental D2Y98P strain (WT), except T209L mutant which displayed significantly smaller plaques (Fig. EV1). To evaluate the mutation stability, the virus mutant strains were serially passaged in mosquito C6/36 cells and their genome was sequenced. Mutants harboring N130Q and T209L substitutions were found stable after 3 passages in C6/36 cells, while the rest reverted to parental glycosylation motif (Appendix Table S1). This observation thus indicated that the nature of the amino acid substitution within the glycosylation motif plays an important role in virus viability.

**Figure EV1:**

Plaque morphology of WT and de-glycosylated NS1 DENV mutants. The viruses were amplified in C6/36 cells and plaqued on BHK-21 cells. Data shown are representative of at least 2 independent biological repeats.

### N130Q and T209L DENV mutants display parental in vitro fitness, while T209L DENV is attenuated in mice

**Figure 1:**
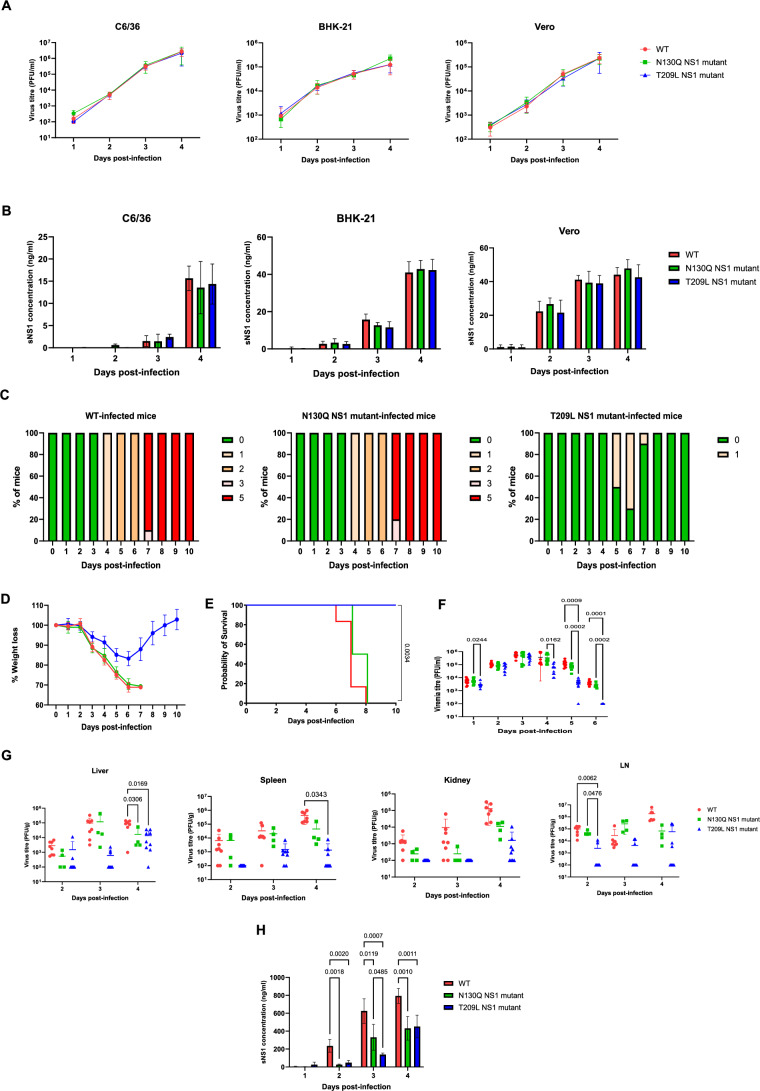
In vitro and in vivo fitness of N130Q and T209L DENV mutants. (**A**) Viral titers in culture supernatants. C6/36, BHK-21, and Vero cells were infected at MOI 0.1 with D2Y98P (WT), N130Q or T209L DENV mutants (*n* = 6; 3 technical replicates per biological replicate; 2 biological repeats). Viral titers in the culture supernatants were measured by plaque assay. The detection limit was set at 10^2^ PFU/mL. (**B**) sNS1 concentration in culture supernatants was determined by ELISA (*n* = 6; 3 technical replicates per biological replicate; 2 biological repeats). (**C**–**G**) IFNAR^−/−^ mice were infected with 10exp6 PFU of each virus via the subcutaneous (sc.) route. (**C**–**E**) Survival, body weight profile and clinical scores (*n* = 10); 0—healthy, 1—ruffled fur, 2—hunched back, 3—lethargy, 4—limb paralysis, 5—mice displaying 30% weight loss (euthanasia). (**F**, **G**) Viral titers in blood (**F**) and perfused organs (**G**) were determined by plaque assay (*n* = 4–9). (**H**) Systemic sNS1 levels were measured by ELISA (*n* = 5). Data shown in panels (**C**) to (**G**) are a combination of 2 biological repeats. All graphs were expressed as mean ± SD. Data analysis were performed using Log-rank (Mantel–Cox) test (**E**) and two-way ANOVA Tukey’s multiple comparisons test (**F**–**H**). Source data are available online for this figure.

The in vitro fitness of N130Q and T209L DENV mutants in mosquito and mammalian cell lines was comparable to that of WT DENV as evidenced by similar viral titer profiles (Fig. 1A) and soluble NS1 (sNS1) levels (Fig. 1B) measured in the culture supernatant. These results thus indicated that either NS1 glycans are dispensable for DENV replication and NS1 secretion in vitro.

We next examined the role of NS1 glycosylation in DENV fitness in a symptomatic mouse model previously established in our laboratory (Tan et al, 2010; Lee et al, 2020). IFNAR^−/−^ mice infected with N130Q mutant displayed clinical manifestations, body weight loss profile and viremia titers that were comparable to mice infected with WT virus, although significantly lower viral titers were measured in perfused liver at day 4 p.i. (Fig. 1C–F). In contrast, mice infected with T209L mutant displayed reduced disease severity as evidenced by milder clinical scores, less than 20% body weight loss and 100% survival (Fig. 1C–E). Furthermore, while the peak viremia titers (day 3 p.i.) in mice infected with T209L mutant were similar to those measured in WT-infected animals, lower titers were measured at days 4, 5 and 6 p.i. (Fig. 1F). In addition, significantly lower viral titers were also measured in perfused organs including the liver and spleen at day 4 p.i. and draining lymph nodes at day 2 p.i. (Fig. 1G), which may also contribute to the attenuated phenotype observed. Interestingly, significantly lower systemic levels of sNS1 were measured in mice infected with either mutant compared to WT control (Fig. 1H), therefore ruling out the hypothesis that lower concentrations of circulating sNS1 account for the attenuated disease severity upon infection with T209L mutant.

Together, these results indicated that N207 glycans on NS1, but not N130 glycans, play a critical role in the fitness and virulence of D2Y98P in IFNAR^−/−^ mice.

### T209L NS1 displays 3D structure, conformation and lipid cargo composition comparable to WT NS1

**Figure 2:**
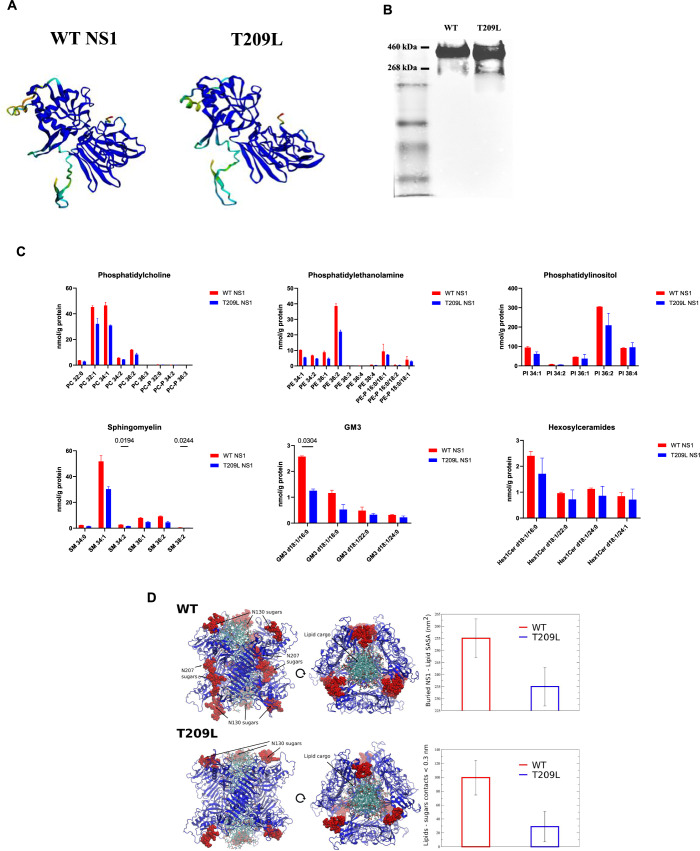
Structural and biochemical features of purified WT and T209L NS1 proteins. (**A**) Simulated 3D structure of monomeric WT and T209L NS1 proteins using AlphaFold2 software. (**B**) Detection of multimeric T209L and WT sNS1 by native PAGE western blot. (**C**) Lipid composition of WT and T209L sNS1 proteins determined by mass spectrometry. (**D**) Representative snapshots from MD simulations of WT and NS1 mutants. Cartoon representation from side (left) and top (right) views. NS1 protein is shown in blue. Lipids are shown in liquorice representation (cyan-carbon, blue-nitrogen, red-oxygen) while glycans are shown as red spheres. The graphs show last 100 ns averaged number of contacts between lipids and glycans as well as NS1:glycan–lipid buried SASA. The buried solvent accessible surface area (SASA) between NS1:glycans and lipid cargo was calculated as a sum of NS1:glycans and lipids before subtracting (NS1:glycan)–lipid SASA. Data shown are representative of at least two independent technical repeats. Data were expressed as mean ± SD. Data analysis were performed using two-way ANOVA Sidak’s multiple comparisons test (**C**). Source data are available online for this figure.

Since T209L substitution on NS1 was found to significantly impact DENV in vivo fitness, we hypothesized that the lack of glycans at N207 may disrupt the conformation and/or lipid composition of multimeric NS1, that may in turn influence some of its biological properties. AlphaFold2 simulation indicated comparable 3D structure between WT and T209L NS1 monomers (Fig. 2A). Furthermore, when purified WT and T209L NS1 proteins were analyzed in native PAGE followed by western blot, a high molecular weight single band (corresponding to multimeric NS1) was detected, although T209L NS1 migrated slightly further than WT NS1, likely due to the lack of glycans at position 207 (Fig. 2B). NS1 lipid cargo composition was analysed by mass spectrometry. Six dominant lipid classes were detected for both WT and T209L NS1 proteins, namely phosphatidylcholine, phosphatidylethanolamine, phosphatidylinositol, sphingomyelin, GM3 and hexosylceramide species (Fig. 2C), as previously described (Gutsche et al, 2011). Lower lipid content was observed with T209L NS1 compared to WT NS1, although the differences did not reach statistical significance for most of the lipid species analysed. Finally, all-atom molecular dynamics (MD) simulations were performed in explicit water using WT or T209L NS1 proteins and their lipid cargo. The glycan motifs at N130 and N207 used in these simulations were those experimentally determined by glycomics and glycoproteomics (Appendix Fig. S1). Since the focus of in silico approach was to study molecular details of protein–sugar interactions, we used weak position restraints on protein alpha carbons to preserve the hexameric assembly in its near experimental conformation. Simulation with WT NS1 indicated many interactions between N130 glycans localized away from the hydrophobic core of the hexamer, near the edges of the protein where lipids could potentially dissociate (Fig. 2D). In contrast glycans on N207 were positioned lower with respect to the hexamer center of mass near the hydrophobic core domain (residues 1–29), with negligible interactions with lipids (Fig. 2D). Therefore, N130 glycans appeared to keep the lipids inside of the hexameric structure as exhibited by buried area between NS1:glycan and lipids. Simulation with T209L NS1 led to similar findings whereby N130 sugars displayed many contacts with lipids which were well-contained inside the hexamer (Fig. 2D).

Together, these observations suggested that the T209L substitution in NS1 minimally affected the structure, lipid composition and stability of hexameric NS1 and further suggested that the in vivo attenuation was not due to compromised structural integrity of T209L NS1 protein.

### T209L NS1 exerts a dominant attenuated phenotype

**Figure 3:**
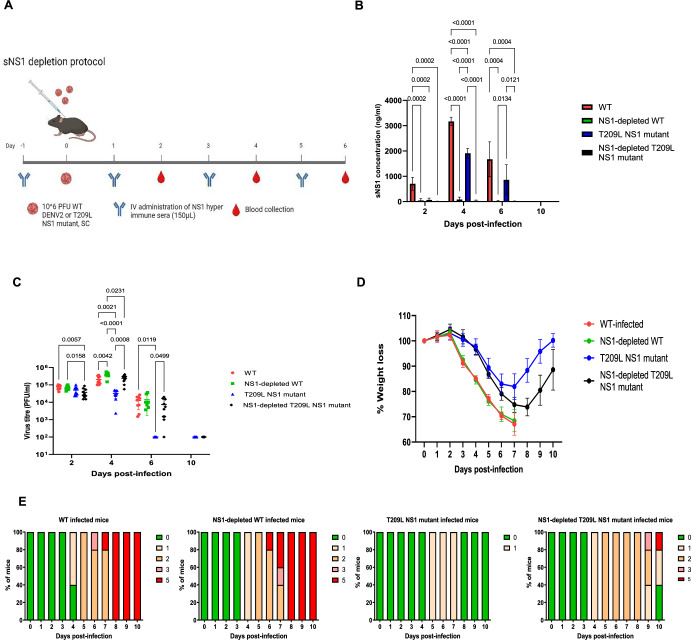
In vivo sNS1 depletion. (**A**) Experimental design of sNS1 depletion in infected IFNAR^−/−^ mice. (**B**) Systemic sNS1 concentrations measured by ELISA (*n* = 9). (**C**) Viremia titers measured by plaque assay (*n* = 10). (**D**) Body weight profile (*n* = 9). (**E**) Clinical scores as described in the Legend of Fig. 1 (*n* = 10). Data shown are a combination of two biological repeats. All graphs were expressed as mean ± SD. Data analysis were performed using two-way ANOVA Tukey’s multiple comparisons test (**B**, **C**). Source data are available online for this figure.

To further investigate the mechanisms involved in the attenuated phenotype observed in mice infected with T209L DENV, we assessed the role of soluble T209L NS1 protein (T209L sNS1) that circulates in the blood during infection. To do so, a depletion experiment was carried out where mice were treated with NS1 hyper-immune serum (Fig. 3A,B). As reported by us before (Lee et al, 2020), sNS1 depletion did not influence disease progression and severity in mice infected with WT D2Y98P virus (Fig. 3C–E), confirming minimal role of circulating sNS1 in dengue pathogenesis in this mouse model. In contrast, sNS1 depletion in T209L DENV-infected mice significantly worsened disease severity, as evidenced by more pronounced body weight loss and increased clinical scores compared to T209L DENV-infected mice, which correlated with significantly higher viremia titers at day 4 and 6 p.i. (Fig. 3C–E). This observation thus indicated that while circulating WT sNS1 did not contribute to the virulent phenotype of WT DENV, T209L sNS1 contributed to the attenuated phenotype of T209L NS1 DENV mutant.

**Figure 4:**
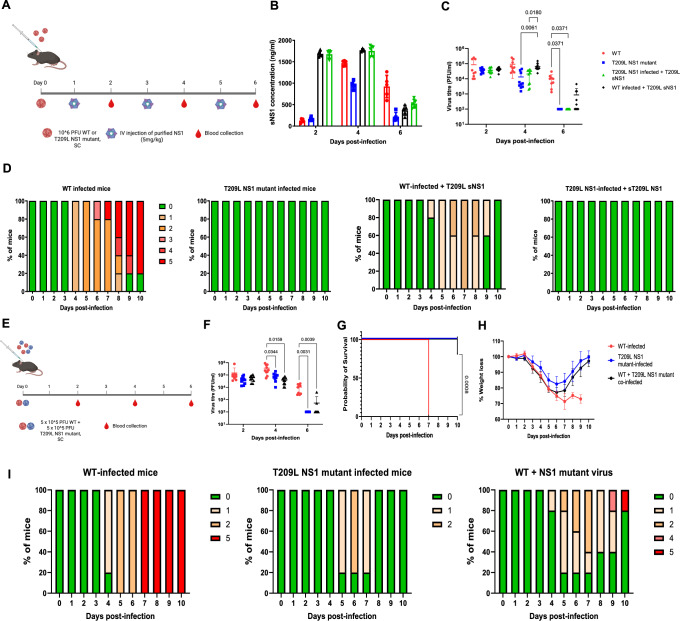
Exogenous administration of sNS1 and co-infection experiments. (**A**–**D**) Exogenous administration of sNS1 in vivo. (**A**) Experimental design. (**B**) Systemic sNS1 concentrations measured by ELISA (*n* = 5 from one biological repeat). (**C**) Viremia titers measured by plaque assay (*n* = 10). (**D**) Clinical scores as described in the legend of Fig. 1 (*n* = 10). (**E**–**I**) Co-infection with WT DENV and T209L DENV. IFNAR^−/−^ mice were sc. infected with either WT or T209L mutant (10^6^ PFU per mouse) or were co-infected with WT and T209L NS1 mutant viruses (5 × 10^5^ PFU of each virus). (**E**) Experimental design. (**F**) Viremia titers were measured by plaque assay (*n* = 10). (**G**) Survival rate (*n* = 10), (**H**) Body weight profile (*n* = 10). (**I**) Clinical scores as described in the legend of Fig. 1 (*n* = 10). 0—healthy, 1—ruffled fur, 2—hunched back, 3— lethargy, 4—limb paralysis, 5—mice displaying 30% weight loss (euthanasia). Data shown in panels (**C**–**I**) are a combination of 2 biological repeats. All graphs were expressed as mean ± SD. Data analysis were performed using two-way ANOVA Tukey’s multiple comparisons test (**C**, **F**) and Log-rank (Mantel–Cox) test (**G**). Source data are available online for this figure.

Conversely, exogenous administration of purified T209L sNS1 in WT-infected mice reduced the disease severity compared to WT-infected controls (Fig. 4A–D). However, the viremia profiles were not significantly different between both groups, although a lower viral titer trend in the T209L sNS1-treated group was clearly observed at day 6 p.i. (Fig. 4C), which likely contributed to the attenuated phenotype observed. In contrast, exogenous administration of T209L sNS1 did not influence the clinical scores and viremia titers in T209L-infected mice (Fig. 4C,D), confirming that the attenuated effect was not due to lower levels of circulating sNS1 in these mice.

Furthermore, a co-infection experiment was performed where equal amounts (5 × 10^5^ PFU) of WT and T209L DENV viruses were co-administered (Fig. 4E–I). Significant disease attenuation was observed in the co-infected mice that phenocopied the attenuation observed with mice infected with T209L DENV only, including milder clinical scores, 100% survival rate, and significantly lower viremia titers at day 4 and 6 p.i. compared to WT-infected controls (Fig. 4E–I). Similar outcome was observed when mice were co-infected with 10exp6 PFU of each virus (Appendix Fig. S2).

Altogether, these data supported that T209L NS1 mutation was dominant over WT NS1 and drives DENV attenuation. We speculated that T209L NS1 mutant protein may modulate some immune responses leading to improved disease outcome.

### The attenuated phenotype of T209L DENV involves T cells

**Figure 5:**
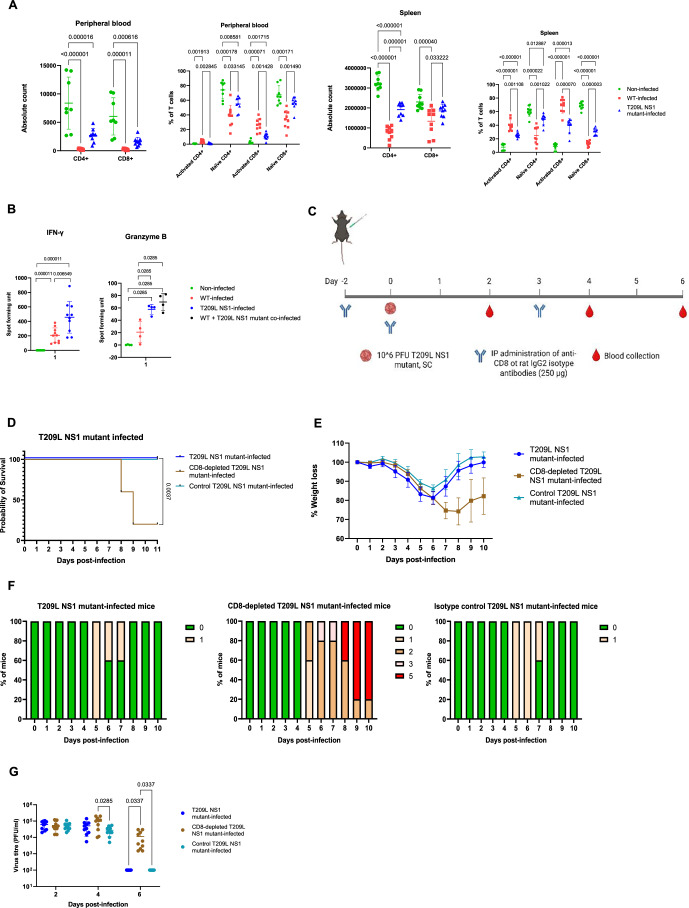
Role of T cells during infection with T209L DENV infection. (**A**) FACS analysis of naive and activated CD4^+^ and CD8^+^ T cell subsets in WT- or T209L DENV-infected IFNAR^−/−^ mice at day 5 p.i. (*n* = 8–9) (**B**) IFNγ-ELISPOT (*n* = 10 from two biological repeats) and granzyme-ELISPOT (*n* = 4 from one biological repeat) of splenic CD8^+^ T cells purified from infected mice at day 3 and 5 p.i. and restimulated with a DENV-specific CD8 immunodominant peptide. (**C**–**G**) CD8^+^ T cell depletion in IFNAR^−/−^ mice infected with T209L NS1 mutant. Data shown are a combination of two biological repeats. (**C**) Experimental design. (**D**) Survival rate (*n* = 10). (**E**) Body weight profile (*n* = 10). (**F**) Clinical scores as described in the legend of Fig. 1 (*n* = 10). (**G**) Viremia titers were determined by plaque assay (*n* = 10). All graphs were expressed as mean ± SD. Data analysis were performed using two-way ANOVA Tukey’s multiple comparisons test (**A**, **G**), nonparametric Multiple Mann–Whitney *t* test (**B**), and Log-rank (Mantel–Cox) test (**G**). Source data are available online for this figure.

The accelerated viral clearance observed from day 4 p.i. onwards in T209L DENV-infected mice (Fig. 1F) prompted us to examine the T cell responses. Indeed, T cells have been reported to play an important role in controlling viral loads and viral clearance, particularly early post-infection when a robust IgG antibody response has yet to be mounted (Lam et al, 2017). We analyzed by flow cytometry the T cell subsets present in the blood and spleen at day 5 p.i. Significantly lower number of total CD4^+^ and CD8^+^ T cells were measured in the blood and spleen from infected mice compared to uninfected controls, indicating lymphopenia in both infected groups (Fig. 5A), and consistent with previous clinical studies (Potts and Rothman, 2008; Wilder-Smith et al, 2004). However, lymphopenia was more pronounced in WT-infected mice as evidenced by lower CD4^+^ and CD8^+^ T cell counts compared to T209L DENV-infected mice (Fig. 5A). When analyzing T-cell subsets, higher levels of naive CD4^+^ and CD8^+^ T cells, and lower levels of activated CD4^+^ and CD8^+^ T cells were consistently found in mice infected with T209L DENV mutant compared to WT-infected animals (Fig. 5A).

Furthermore, to assess the functionality of live T cells, CD8^+^ T cells were purified from the spleen of WT- and T209L DENV-infected mice and were re-stimulated with a DENV-specific immunodominant CD8^+^ T cell epitope (Yauch et al, 2009). Results indicated that at day 5 p.i. the number of IFN-γ and granzyme-producing CD8^+^ T cells was significantly greater in spleens from T209L DENV-infected and co-infected mice, compared to WT-infected controls (Fig. 5B).

Since CD8^+^ T cells have been shown to play an important role in viral clearance during primary dengue (Yauch et al, 2009) while CD4^+^ T cells were dispensable (Yauch et al, 2010), a CD8^+^ T-cell depletion experiment was conducted to further evaluate the role of this cell subset during infection with WT or T209L DENV mutant. CD8^+^ T-cell depleted animals displayed increased disease severity as evidenced by increased mortality rate and increased clinical scores, which correlated with increased viremia titers (Fig. 5C–G; Appendix Fig. S3). This result thus confirmed the protective role of CD8^+^ T cells in our symptomatic DENV2 mouse model and indicated that the attenuated phenotype of T209L DENV2 depends on CD8^+^ T cells.

Finally, to exclude a role of antibodies in the attenuated phenotype observed with T209L DENV, we infected IFNAR-muMT mice that are type I IFN receptor- and B-cell deficient (Ting et al, 2025). Results showed that T209L DENV remained attenuated in this mouse model, with milder clinical scores and accelerated viral clearance compared to WT-infected controls (Fig. EV2).

**Figure EV2:**

Infection profile of WT and T209L DENV in IFNAR-muMT mice. (**A**) Body weight profile (*n* = 9). (**B**) Viremia titers were determined by plaque assay (*n* = 4–9). (**C**) Clinical scores as described in the legend of Fig. 1 (*n* = 9). Data shown are a combination of 2 independent repeats. All graphs were expressed as mean ± SD. Data analysis were performed using two-way ANOVA Sidak’s multiple comparisons test (**B**).

Together, these observations supported that the attenuated phenotype observed in mice infected with T209L DENV mutant was driven by T cells, possibly through more effective viral clearance by CD8^+^ T cells.

### T209L DENV down-modulates the host early inflammatory response

**Figure 6:**
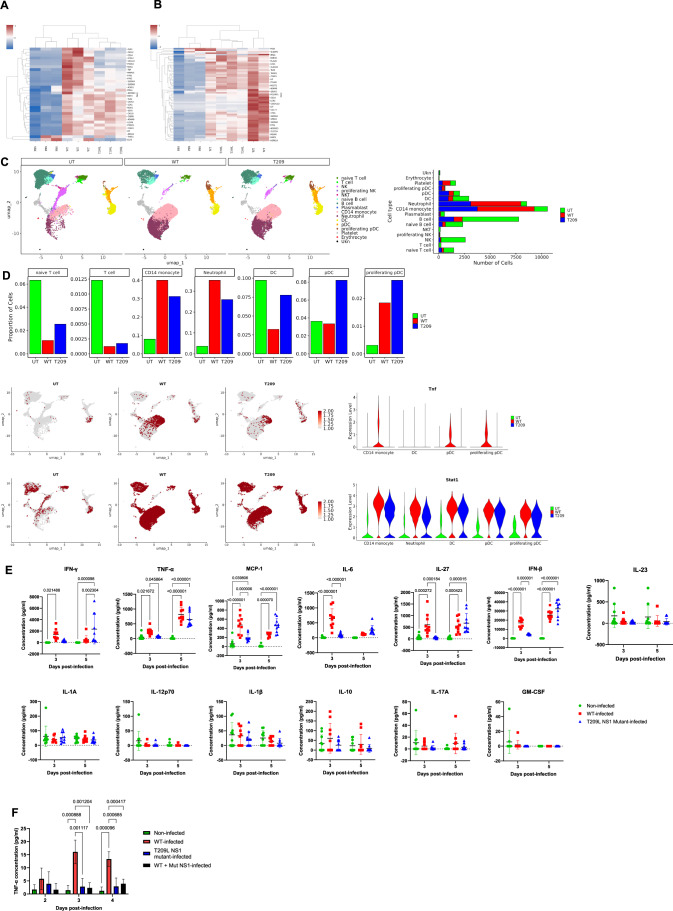
Inflammatory responses in response to infection with WT or T209L DENV. Bulk (**A**, **B**) and single cell (**C**, **D**) RNA sequencing on white blood cells harvested at day 3 p.i. from mice infected with WT DENV, T209L DENV or left uninfected (*n* = 3, one biological repeat). (**A**) Innate immune responses and inflammatory responses (Reactome). (**B**) Expression levels of genes related to neutrophil activation and degranulation. (**C**) Annotated UMAP. (**D**) TNF-α and STAT1 gene expression. (**E**) Systemic cytokine profile measured by ELISA in mice infected with WT or T209L DENV mutant at day 3 and 5 p.i. (*n* = 10 from two biological repeats). (**F**) THP-1 macrophages were infected with WT, T209L DENV or a mix of both (1:1). TNFα levels in culture supernatants were measured by ELISA (*n* = 6 from two biological repeats). All graphs were expressed as mean ± SD. Data analysis were performed using two-way ANOVA Tukey’s multiple comparisons test (**E**, **F**). Source data are available online for this figure.

**Figure EV3:**
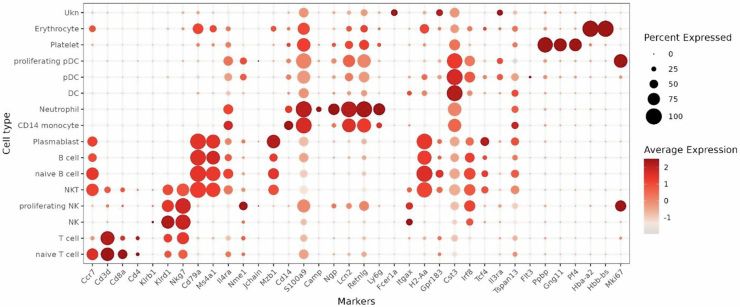
Relative abundance of markers of immune cell clusters used for single cell RNAseq data analysis.

To understand the upstream mechanisms that drive the improved T-cell responses in T209L DENV-infected mice, comparative bulk (RNAseq) and single cell RNAseq (scRNAseq) transcriptomics approaches were carried out. Whole blood samples from mice infected with WT or T209L DENV were harvested at day 3 p.i., a time point at which comparable viremia titers were observed in both infected groups (Fig. 1F). Cluster analysis of bulk RNAseq data revealed that infection with WT DENV displayed greater innate immune activation and stronger inflammatory response (Fig. 6A) with a pronounced neutrophil activation signature (Fig. 6B), compared to mice infected with T209L DENV. To dissect further the immune cells involved in those transcriptional changes, we performed a single cell RNAseq experiment, which revealed differential cell subset gene frequencies between infected and uninfected controls that supported lymphopenia (B and T cells), reduced number of NK cells, and increased number of monocytes, neutrophils, and dendritic cells (Figs. 6C and  EV3) in both infected groups. When comparing both infected groups, mice infected with T209L DENV displayed greater number of plasmacytoid DCs and naive T cells, but lower number of monocytes and neutrophils compared to WT-infected mice (Fig. 6C). Next, to examine the differences in inflammatory responses at the single-cell level, we probed for the expression of TNFα and STAT1 in the different immune cell subsets. Greater TNFα and STAT1 gene expression were observed in monocytes, DCs and neutrophils from WT-infected mice compared to animals infected with T209L DENV mutant (Fig. 6D). Consistent with the transcriptomic signals, higher systemic levels of IFN-γ, TNF-α, IL-6, MCP-1 and IFNβ were measured by ELISA at day 3 p.i. in mice infected with WT DENV compared to mice infected with T209L DENV (Fig. 6E). At day 5 p.i. however, comparable cytokine levels between both infected groups were detected, except for IFNγ that was significantly higher in mice infected with T209L DENV mutant, which was consistent with the increased number of IFNγ-producing CD8^+^ T cells in this infected group (Fig. 5B). These pro-inflammatory cytokine kinetic profiles were consistent with the observation that vascular leakage in liver and kidneys was more pronounced in WT-infected animals at day 4 p.i. but not at day 6 p.i., compared to T209L DENV-infected group (Appendix Fig. S4).

Together, both RNAseq analyses and systemic cytokine profiles supported the hypothesis that T209L NS1 protein down-modulates or delays the initial host inflammation response during infection, which in turn influences favourably disease outcome through reduced vascular leakage and improved T-cell mediated viral clearance. Consistently, human THP-1 macrophages infected with T209L DENV mutant produced significantly less TNFα than WT-infected cells (Fig. 6F). Co-infection with both viruses led to similarly lower TNFα levels, confirming the dominant attenuating effect of T209L DENV mutant.

### T209L NS1 influences T cell fate via the PD-L1/PD-1 axis

**Figure 7:**
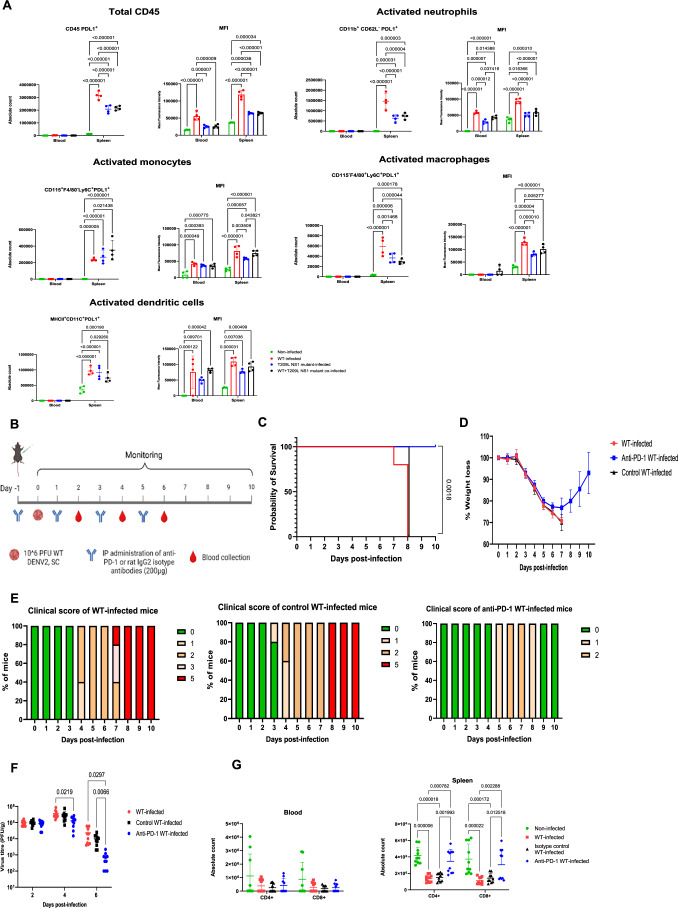
PD-L1 expression on innate immune cells and PD-1 blocking experiment. (**A**) Flow cytometric detection of PD-L1^+^ circulating and splenic total CD45^+^ cells, activated neutrophils, activated monocytes, activated macrophages and activated dendritic cells harvested at day 3 p.i. from WT-, T209L- and co-infected IFNAR^−/−^ mice (*n* = 4 from one biological repeat). Absolute counts and mean fluorescence intensity (MFI) of PD-L1 expression are shown. (**B**–**G**) PD-1 blockade experiment. (**B**) Experimental design. (**C**) Survival rate (*n* = 10). (**D**) Body weight profile (*n* = 8). (**E**) Clinical scores as described in the legend of Fig. 1 (*n* = 10). (**F**) Viremia titers were measured by plaque assay (*n* = 10). (**G**) CD4^+^ and CD8^+^ T cell counts in blood and spleen harvested at day 5 p.i. were analysed by flow cytometry (*n* = 9–10). Data shown in (**C**–**G**) are a combination of two biological repeats. All graphs were expressed as mean ± SD. Data analysis were performed using two-way ANOVA Tukey’s multiple comparisons test (**A**, **F**, **G**) and Log-rank (Mantel–Cox) test (**C**). Source data are available online for this figure.

**Figure EV4:**
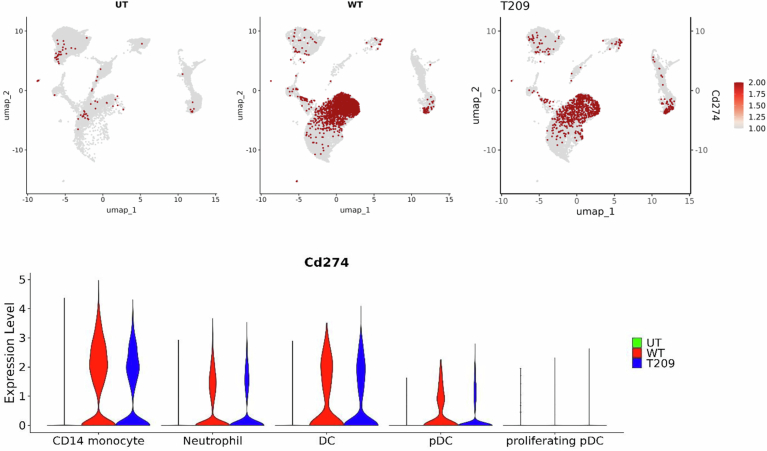
PD-L1 (CD274) gene expression in neutrophils, monocytes and dendritic cells in DENV-infected mice. The expression of PD-L1 gene in monocytes, neutrophils and dendritic cell subsets in WT- and T209L DENV-infected IFNAR^−/−^ mice was determined by single cell RNAseq.

**Figure EV5:**
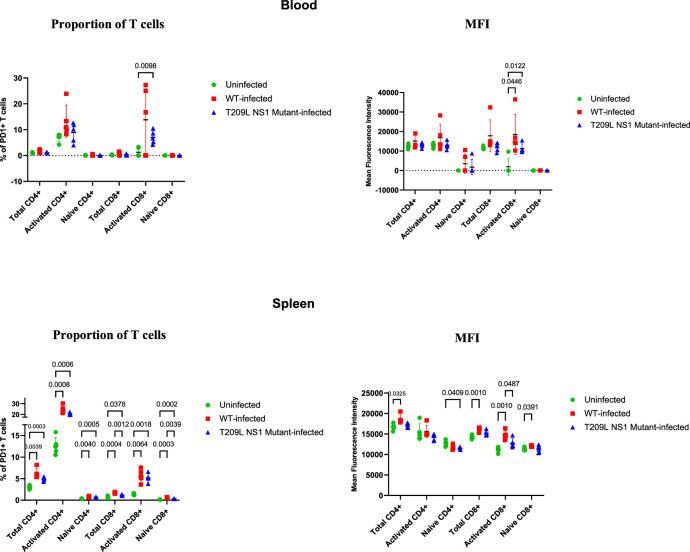
PD-1 expression on T cells. Flow cytometry analysis of circulating and splenic T cells harvested at day 3 p.i. from WT- or T209L NS1 DENV-infected IFNAR^−/−^ mice (*n* = 5). Percentages of PD-1^+^ T cells and mean fluorescence intensity (MFI) of PD-1 expression on T cells. Data shown are from one biological repeat. All graphs were expressed as mean ± SD. Data analysis were performed using two-way ANOVA Tukey’s multiple comparisons test.

The combined observations of blunted early inflammatory responses and improved DENV-specific CD8^+^ T cell response in mice infected with T209L DENV mutant prompted us to probe the involvement of the PD-L1/PD-1 axis in disease severity. Indeed, during infection innate immune cells such as neutrophils, dendritic cells and monocytes have been shown to engage with T cells through PD-L1/PD-1 interactions, leading to T-cell apoptosis and lymphopenia (de Kleijn et al, 2013; Francisco et al, 2010; Garcia-Diaz et al, 2017; Jiang et al, 2019; Sun et al, 2018). Furthermore, studies have reported that PD-L1 expression on innate immune cells is modulated by TNF-α, IL-6 and IFN-γ (Bardhan et al, 2016; de Kleijn et al, 2013; Francisco et al, 2010; Jiang et al, 2019; Wilke et al, 2011). Hence, we hypothesized that the lower levels of inflammatory cytokines measured early post-infection in mice infected with T209L DENV may lead to reduced PD-L1 expression on innate immune cells, thereby limiting engagement with T cells through the PD-L1/PD-1 axis and preventing or minimizing T-cell death. Consistent with this idea, increased PD-L1 gene (Cd274) expression in neutrophils, monocytes and dendritic cells (DC) was observed in WT-infected mice compared to mice infected with T209L DENV mutant (Fig. EV4). In addition, increased counts of PD-L1^+^ total leukocytes (CD45^+^), and activated innate immune cell subsets including neutrophils, monocytes, macrophages and DCs combined with increased PD-L1 mean fluorescence intensity (MFI) signals were measured by flow cytometry in WT-infected mice compared to the T209L DENV-infected group (Fig. 7A). Of note, mice co-infected with WT and T209L DENV displayed comparable trends as the mice infected with T209L mutant alone (Fig. 7A), further supporting a dominant suppressive effect by T209L mutant. Finally, a trend in increased PD-1 expression was also observed on both circulating and splenic activated CD4^+^ and CD8^+^ T cells in WT-infected mice compared to the mutant-infected group, although the differences did not reach statistical significance (Fig. EV5).

To further examine the involvement of the PD-L1/PD-1 axis in dengue disease severity, we evaluated whether blocking PD-L1/PD-1 interactions with a PD-1 monoclonal antibody (mAb) influences severe disease outcome (Fig. 7B–G). The results indicated that treatment with PD-1 mAb effectively protected mice from severe disease, as evidenced by 100% survival and milder clinical manifestations compared to infected only and isotype-treated control groups (Fig. 7C–E). This improved disease outcome correlated with lower viremia titers at day 4 and 6 p.i. (Fig. 7F), and increased levels of both CD4^+^ and CD8^+^ T cells in the spleen from PD-1 mAb treated mice (Fig. 7G).

Together, these observations thus supported that the lower levels of PD-L1 and PD-1 expression in mice infected with T209L DENV reduced T-cell apoptosis, which improved viral clearance. Conversely, in WT-infected mice, increased PD-L1 expression on innate immune cells and higher PD-1^+^ T cells enhanced T cell death through PD-L1/PD-1 interactions, and impaired T-cell mediated viral clearance.

## Discussion

Previous in vitro studies have reported variable effects of flavivirus NS1 mutants that lacked glycans on either N130 or N207. Mutation at N130 on DENV4 NS1 led to reduced viral growth in mammalian and mosquito cells (Pletnev et al, 1993), whereas the same mutation did not affect viral production of DENV2 16681 strain in mammalian cells (Crabtree et al, 2005). Abrogation of N207 glycosylation site on DENV1 and DENV2 NS1 did not attenuate the viruses in mammalian cells although a delayed cytopathic effect was noted (Crabtree et al, 2005; Tajima et al, 2008); while in vitro attenuation was reported with N207 de-glycosylated NS1 expressed by DENV2 NGC strain (Pryor and Wright, 1994). Here, we did not observe any significant in vitro attenuation when either N-glycosylation site was abrogated on NS1 from D2Y98P virus, a representative isolate of Cosmopolitan DENV2 strains that have prevailed in SEA for several decades and have caused recurrent outbreaks (Yenamandra et al, 2021). These observations thus suggested that the role and importance of NS1 glycans at N130 and N207 in DENV in vitro fitness depend on the DENV serotype and genotype.

In vivo, while N130 mutation has been reported to attenuate DENV2 16681 and DENV4 virulence in suckling mice (Crabtree et al, 2005; Fang et al, 2023; Pletnev et al, 1993), the loss of the glycans at this site did not influence D2Y98P virus fitness and virulence in IFNAR^−/−^ adult mice. In contrast, glycans at position N207 were found to play a critical role in D2Y98P virulence, and this observation was consistent with previous studies using another DENV2 strain (Crabtree et al, 2005; Pryor and Wright, 1994). In those earlier studies, the reduced virulence observed was proposed to be linked to the reduced levels of soluble NS1 in circulation. However, although we also detected lower levels of sNS1 in mice infected with T209L DENV mutant, a similar reduction in circulating sNS1 levels was measured in mice infected with N130Q DENV mutant, the latter did not result in attenuation. Hence, we concluded that the attenuated phenotype of T209L DENV could not be attributed to lower sNS1 levels in circulation. Instead, our data indicated that T209L NS1 exerted a dominant attenuating effect over WT NS1 protein. Several lines of evidence supported this claim. Firstly, antibody-mediated depletion of T209L sNS1 in circulation worsened disease severity in mice infected with T209L DENV mutant, while depletion of WT sNS1 did not attenuate WT infection, as we previously reported (Lee et al, 2020). Secondly, exogenous administration of purified T209L sNS1 in mice infected with WT DENV led to significant disease attenuation. Thirdly, co-infection with WT and T209L DENV mutant phenocopied the attenuated phenotype seen with mice infected with T209L DENV alone.

We next explored the mechanisms of attenuation observed in mice infected with T209L DENV. Our data supported that during infection with this mutant, interactions between innate immune cells and T cells through the PD-L1/PD-1 axis were reduced, hence limiting T-cell apoptosis and improving T cell-mediated viral clearance. In support of this model, reduced PD-L1 gene expression on innate immune cells and less PD-1^+^ T cells were measured in mice infected with T209L DENV compared to WT-infected mice. Consistent with this observation, less pronounced lymphopenia and more functional DENV-specific CD8^+^ T cells were observed in mice infected with T209L DENV mutant. Furthermore, CD8^+^ T cell depletion in mice infected with T209L DENV worsened disease severity, confirming the protective role of CD8^+^ T cells in this attenuated infection model, while the role of B cells and antibodies was ruled out in our newly established IFNAR-muMT infection model (Ting et al, 2025). Finally, involvement of the PD-L1/PD-1 axis in dengue disease severity was directly demonstrated by treating WT-infected mice with an anti-PD-1 blocking mAb, which resulted in complete protection from lethal outcome, accelerated viral clearance and reduced lymphopenia, phenocopying the infection outcome seen in mice infected with T209L DENV.

Our findings are in line with a recent in vitro study that conducted in-depth phenotypic characterization of the PBMCs obtained from severe dengue patients compared to non-severe dengue patients (Gregorova et al, 2025). In that study, increased PD-1^+^ expression and decreased granzyme production were observed in CD8^+^ T cells from severe dengue patients, and in vitro PD-1/PD-L1 blockade increased T cell polyfunctionality and CD8^+^ T cells cytotoxic potential (Gregorova et al, 2025). Other studies have investigated the role of PD-L1/PD-1 axis in the impairment of T cells function during viral infection and the potential importance of rescuing these T cells to improve disease outcome, including Hepatitis C virus (HCV), human metapneumovirus (HMPV), influenza virus, simian immunodeficiency virus (SIV) and human immunodeficiency virus (HIV) (Urbani et al, 2006; Erickson et al, 2012; McNally et al, 2013; Rutigliano et al, 2014; Velu et al, 2009; Palmer et al, 2013). However, to our knowledge this is the first time that the PD-L1/PD-1 axis is demonstrated to play a critical role in dengue pathogenesis.

We propose that the limited engagement of the PD-L1/PD-1 axis during infection with T209L DENV is mainly driven by a blunted pro-inflammatory response early post-infection whereby lower levels of key cytokines including IL-6, IFN-γ and TNF-α limit PD-L1 upregulation on a variety of innate immune and non-immune cells (Bardhan et al, 2016; de Kleijn et al, 2013; Francisco et al, 2010; Jiang et al, 2019; Wilke et al, 2011). We hypothesize that T209L NS1 protein actively down-modulates the production of these pro-inflammatory cytokines. In support of this hypothesis, the blunted inflammatory response and lower inflammatory cytokine levels measured in mice infected with T209L DENV cannot be explained by lower viral titers since these observations were made at a time point where viral titers were comparable to those measured in WT-infected mice. Furthermore, we showed that infection with T209L DENV of THP-1 macrophages led to reduced TNFα levels compared to WT infection. A previous study has reported that fully glycosylated DENV NS1 protein could signal through TLR4 and induce the production of pro-inflammatory cytokines (Modhiran et al, 2015). However, since the T209L NS1 attenuated phenotype is dominant over WT, it cannot be explained by the loss of interaction with TLR4. Hence, the mechanism by which T209L NS1 down-modulates pro-inflammatory responses remains to be uncovered. Nevertheless, our findings imply that in the context of WT DENV infection, glycans at N207 prevent NS1 from exerting this down-modulatory effect, which represents a novel immune evasion strategy.

In conclusion, our study has unraveled a novel immune evasion strategy by DENV that implicates N207 glycans on NS1, and a novel immunopathogenesis mechanism in severe dengue that involves the PD-L1/PD-1 axis, resulting in premature T-cell apoptosis, which impairs viral clearance. The highly conserved N-glycosylation sites on NS1 across flaviviruses strongly suggest that our findings may be extended to the entire family, and mutating these sites could be considered for the development of next generation live attenuated DENV vaccine strains. Furthermore, and importantly, our work supported that targeting the PD-L1/PD-1 axis could represent a promising host targeted therapeutic approach to limit T cell death and improve viral clearance in severe dengue patients.

## Methods

**Table:** Reagents and tools table

Reagent/resource	Reference or source	Identifier or catalog number
**Mammalian cell lines**		
C6/36	ATCC	CRL-1660
BHK-21	ATCC	CCL-10
Vero	ATCC	CCL-81
THP-1	ATCC	TIB-202
**DENV strains**		
D2Y98P	Tan et al, 2010	GenBank JF327392.1
N130Q D2Y98P	This study	–
T209L D2Y98P	This study	–
**Mice**		
IFNAR^−/−^	Jackson lab	JAX 029098
IFNAR-muMT	Ting et al, 2025	–
**Proteins/peptides**		
Hexameric NS1 from D2Y98P	The Native Antigen Company	Custom-made
Ovalbumin	InvivoGen	9006-59-1
DENV2 NS4B_99-107_ peptide	Mimotopes	Custom-made
**Antibodies**		
Anti-CD8 antibody (depletion experiment)	BioXCell	BE0061
Rat IgG2b isotype control (depletion experiment)	BioXCell	BE0090
Anti-PD-1	BioXCell	BE0146
Anti-CD45-BUV395 (1:500 dilution)	BD Biosciences	564279
Anti-CD4-BUV805 (1:500 dilution)	BD Biosciences	612900
Anti-CD8α-BV786 (1:500 dilution)	BD Biosciences	752635
Anti-CD44-BUV737 (1:500 dilution)	BD Biosciences	612799
Anti-MHC class II-BV510 (1:200 dilution)	BD Biosciences	743871
Anti-CD11c-BV786 (1:200 dilution)	BD Biosciences	563735
Anti-CD62L-BV605 (1:500 dilution)	BD Biosciences	563252
Anti-Ly6G-PE (1:500 dilution)	Biolegend	127607
Anti-CD11b-BV650 (1:500 dilution)	Biolegend	101239
Anti-PD-1-FITC (1:400 dilution)	Biolegend	135214
Anti-F4/80-BV421 (1:200 dilution)	Biolegend	123131
Anti-CD115-PE (1:200 dilution)	Biolegend	165004
Anti-Ly6C-APC/Cyanine7 (1:200 dilution)	Biolegend	128026
Anti-PD-L1-PE-Cy7 (1:400 dilution)	Biolegend	124314
DENV2 NS1 polyclonal antibody	ThermoFisher Scientific	PA5-32207
HRP-conjugated goat anti-rabbit IgG antibody	BioRad	1706515
mouse IFNγ ELISPOT	BD Biosciences	551083
Granzyme B Monoclonal Antibody (16G6)	eBioscience	14-8822-82
Granzyme B Monoclonal Antibody, Biotin	eBioscience	13-8822-82
BD ELISPOT AEC substrate	BD Biosciences	551951
**Molecular biology kits**		
QIAamp Viral RNA	Qiagen	52904
GoScript™ Reverse Transcriptase	Promega	A5003
NEB Q5 Hot-Start high-fidelity 2× Master Mix	New England Biolabs	M0494S
pCR™-Blunt II-TOPO™ Vector	Invitrogen	K280002
MinElute gel extraction Kit	Qiagen	28604
NEBuilder HiFi DNA Assembly Master Mix	New England Biolabs	E2621X
**Cytokine detection kits**		
LEGENDplex Mouse Inflammatory Panel kit	BioLegend	740446
TNFα	Invitrogen	BMS607-3
Anti-NS1 Mab62.1	Prof C. Ruedl (NTU)	–
Anti-NS1 Mab56.2	Prof Vasudevan (Duke-NUS)	Rozen-Gagnon et al, 2012
**Other reagents**		
Monophosphoryl lipid A (MPLA)	InvivoGen	Vac-mpla
AddaVax	InvivoGen	Vac-adx-10
Primers (Table S2)		Custom-made
phorbol 12-myristate 13-acetate (PMA)	ThermoFisher Scientific	356150010
Evan’s blue	Merck	E2129
Sera-Mag carboxylated speedbeads	Cytiva, Marlborough, MA	45152105050250
**RNAseq experiments**		
RNeasy mini kit	Qiagen	74104
BD Pharm Lyse™ Lysing Buffer	BD Biosciences	555899
Dead Cell Removal kit	Miltenyi Biotec	130-090-101
Chromium Fixed RNA Kit, Mouse Transcriptome	10x Genomics	PN-1000495
Chromium Next GEM Single Cell Fixed RNA Sample Preparation kit	10x Genomics	PN-1000414
Chromium Next GEM Chip Q Single Cell kit	10x Genomics	PN-1000422
Dual Index Kit TS Set A	10x Genomics	PN-1000251
High Sensitivity DNA Kit	Agilent	5067-4626

### Cell lines and viruses

C6/36 *Aedes albopictus* cell line (ATCC; CRL-1660) was maintained in Leibotvitz’s L-15 medium (Gibco) supplemented with 10% FBS (Gibco) at 28 °C. Baby hamster kidney-21 (BHK-21; ATCC; CCL-10) cell line was maintained in RPMI 1640 (Gibco) supplemented with 10% FBS and cultured at 37 °C with 5% CO_2_. African green monkey kidney epithelial (Vero; ATCC; CCL-81) cell line was maintained in DMEM (Gibco) supplemented with 10% FBS and cultured at 37 °C with 5% CO_2_. THP-1 (ATCC; TIB-202) cell line was maintained in DMEM (Gibco) supplemented with 10% FBS and 0.05 mM 2-mercaptoethanol and cultured at 37 °C with 5% CO_2_. Differentiation of THP-1 into macrophage-like cells was done by incubating the cells with 100 ng/mL of phorbol 12-myristate 13-acetate (PMA) for 2 days. All cell lines were routinely tested for mycoplasma contamination using a detection kit (InvivoGen, cat. # rep-mys).

DENV2 (Dengue D2Y98P; GenBank accession no. JF327392.1) derives from a 1998 Singapore clinical isolate that had been exclusively passaged in C6/36 cells and plaque purified twice in BHK-21 cells, before an infectious clone was made. DENV stock was propagated in C6/36 cell line maintained in Leibovitz’s L-15 medium supplemented with 2% FBS as previously described (Lee et al, 2020). Harvested culture supernatants containing the virus particles were stored at −80 °C. Virus titers were determined by plaque assay in BHK-21 cells as described below.

### DENV infection of cell lines

C6/36, BHK-21, and Vero cells were infected at a multiplicity of infection (MOI) of 0.1 while differentiated THP-1 cells were infected at MOI 10 with D2Y98P (WT DENV), N130Q DENV or T209L DENV mutant. Plates were incubated at 37 °C for 1 h with rocking every 15 min for viral adsorption. Each well was rinsed twice with PBS before addition of 200 µl of respective media containing 2% FBS. The plates were incubated for 4 days at 28 °C (C6/36) or 37 °C (BHK-21, Vero, and differentiated THP-1), and the culture supernatants were collected at indicated time points post-infection (p.i.). Viral quantification was performed by plaque assay.

### Virus quantification by plaque assay

Virus titer was quantified by plaque assay in BHK-21 cells as previously described (Lee et al, 2020). Briefly, 45,000 cells/well were seeded in 24-well plates (Nunc) 1 day before plaque assay. Cell monolayers were then infected with 10-fold serially diluted viral suspensions in RPMI 1640 supplemented with 2% FBS. After 1-h incubation at 37 °C with CO_2_, overlay medium [RPMI 1640 containing 1% (wt/v) carboxymethyl cellulose and 2% FBS] was added to each well. After incubation for 4 days at 37 °C with CO_2_, cells were fixed with 4% paraformaldehyde (Sigma-Aldrich) and stained with 0.05% crystal violet (Sigma-Aldrich). Plaques were counted, adjusted by dilution, and expressed as the number of plaque forming units per milliliter (PFU/mL).

### Generation of de-glycosylated NS1 DENV mutants

D2Y98P RNA genome was extracted using QIAamp Viral RNA Kits (Qiagen). Complementary DNA (cDNA) synthesis was performed using GoScript™ Reverse Transcriptase (Promega) as per manufacturer’s instructions. Four PCR fragments of around 2700 nucleotides long were generated from cDNA using primer pairs (Table S2) with NEB Q5 Hot-Start high-fidelity 2× Master Mix (New England Biolabs). Fragments were gel-purified with MinElute gel extraction Kit (Qiagen) after agarose gel electrophoresis and blunt end cloning was performed into pCR™-Blunt II-TOPO™ Vector (Invitrogen). Site-directed mutagenesis was performed on plasmid that carried E gene to introduce the desired mutation using primer pairs (Table S2). The mutated gene was amplified with other genes fragments via polymerase chain reaction (PCR) and seamlessly assembled with vector containing cytomegalovirus (CMV) promoter sequence, hepatitis delta virus (HDV) ribozyme and simian virus 40 (SV40) poly-A sequence using NEBuilder HiFi DNA Assembly Master Mix (New England Biolabs) at 50 °C for 60 min. This CMV-DENV genome-HDVr-SV40pA assembled product was transfected into BHK-21 cells using Lipofectamine 2000 (Invitrogen). Four to six days later, the viral supernatant was collected and sequenced using Sanger sequencing.

### Mouse infection with DENV

5–6-wk-old IFNAR^−/−^ mice were infected subcutaneously (sc.) with 10^6^ PFU of D2Y98P (WT) or NS1 mutants (N130Q or T209L DENV) viruses. 5–6-wk-old IFNAR-muMT mice (Ting et al, 2025) were infected subcutaneously (sc.) with 10^4^ PFU of D2Y98P (WT) or T209L NS1 DENV mutant. Mice were monitored daily for body weight loss and for clinical manifestations. Mice were promptly euthanized when they lost 30% of the body weight measured at the start of the infection experiment. In the co-infection experiment, equal amounts (5 × 10^5^ or 10^6^ PFU) of WT and T209L DENV mutant were mixed and administered sc. to IFNAR^−/−^ mice.

### sNS1 depletion in vivo experiment

In all, 5–6-week-old IFNAR^−/−^ mice were immunized intraperitoneally (ip.) three times (weeks 0, 2, and 6) with 20 µg of purified hexameric NS1 from WT strain (custom-made, The Native Antigen Company) or OVA protein (InvivoGen) adjuvanted with 1 µg of monophosphoryl lipid A (MPLA; InvivoGen) and 1:1 volume AddaVax (InvivoGen) as described previously (Beatty et al, 2015). NS1 immune serum was collected 2 weeks after the third immunization and heat inactivated at 56 °C for 30 min before storage at −80 °C. Antibody titers of immune serum were quantified by ELISA. This NS1 hyper-immune serum was used for passive transfer experiment into IFNAR^−/−^ mice. One day after i.p. administration of NS1 immune serum (150 µl per mouse), IFNAR^−/−^ mice were sc. infected with 10^6^ PFU of WT or T209L DENV mutant. A second and third administration of NS1 hyper-immune serum were performed at day 3 and 5 post-infection (p.i.).

### Exogenous administration of NS1 protein to DENV-infected mice

In all, 5–6-week-old IFNAR^−/−^ mice were infected sc. with 10^6^ PFU of WT or T209L DENV. In total, 5 mg/kg of purified NS1(WT or de-glycosylated T209L) (custom-made, The Native Antigen Company) or OVA protein (InvivoGen) was administered intravenously (iv.) to the infected mice at day 1, 3 and 5 p.i.

### Vascular leakage assessment

Vascular leakage was assessed by the Evan’s blue method as described previously (Lee et al, 2020). Briefly, 5–6-week-old IFNAR^−/−^ mice were infected sc. with 10^6^ PFU of WT or T209L DENV. At day 4 or day 6 p.i., Evans blue (10 μl/g; 0.1% w/v in PBS) was administered intravenously (iv.). Two hours later, mice were euthanized, perfused thoroughly with PBS, and the liver, spleen and kidneys were harvested. Evans blue dye was extracted from the organs using N,N-dimethylformamide at 4 ml/g of tissue wet weight. After centrifugation, the supernatants’ absorbance was read at OD_620nm_ (Mabtech).

### CD8^+^ T cells depletion in DENV-infected mice

In all, 5–6-week-old IFNAR^−/−^ mice were infected sc. with 10^6^ PFU of T209L DENV. Anti-CD8 antibody (BioXCell) or rat IgG2b isotype control (BioXCell) were administered i.p. (250 μg per dose) to the infected mice 2 days prior to infection, and at day 0 and 3 p.i.

### PD-1 blockade

In all, 5–6-week-old IFNAR^−/−^ mice were infected sc. with 10^6^ PFU of WT DENV. In total, 200 μg of anti-PD1 antibody (BioXCell) or rat IgG2b isotype control (BioXCell) was administered i.p. to the infected mice one day prior to infection, and at day 1, 3 and 5 p.i.

### ELISAs

Systemic levels of soluble NS1 (sNS1) in mice were quantified via sandwich ELISA as described previously (Lee et al, 2020). Mouse anti-NS1 Mab62.1 diluted in PBS was coated at 0.1 µg/well onto 96-well enzyme immunoassay plates overnight at 4 °C. After washing and blocking as described above, diluted serum samples (from 1:100 to 1:2500) were added to the wells and incubated for 2.5 h at 37 °C. A standard curve was established by twofold serial dilution of recombinant NS1 (D2Y98P) from 25 to 0.39 ng/ml. Plates were washed five times before the addition of HRP-conjugated mouse anti-NS1 Mab56.2 (25 ng/well) (Rozen-Gagnon et al, 2012), made by conjugating HRP to Mab56.2 using NH_2_ peroxidase labeling kit (Abnova) and incubated for 1.5 h at room temperature. After the final washes, detection was performed with the addition of tetramethylbenzidine (R&D Systems) for 30 min at room temperature. The reaction was stopped with 2 N H_2_SO_4_. Absorbance was read at 450 nm. The concentration of NS1 was calculated based on the standard curve and expressed as the concentration of NS1 in nanograms per milliliter (ng/mL).

### Detection of soluble inflammatory mediators

The systemic levels of cytokines and soluble mediators in uninfected and DENV-infected mice were quantified using LEGENDplex Mouse Inflammatory Panel kit (BioLegend) according to the manufacturer’s protocol. Samples were run using Attune Nxt flow cytometer and analyzed using FlowJo software.

Soluble TNF-α level in infected differentiated THP-1 was quantified using a commercial ELISA kit (Invitrogen) according to the manufacturer’s protocol. The absorbance was read using a spectrophotometer (TECAN).

### Flow cytometry

Anti-CD45-BUV395, anti-CD4-BUV805, anti-CD8α-BV786, anti-CD44-BUV737, anti-MHC class II-BV510, anti-CD11c-BV786, and anti-CD62L-BV605 were purchased from BD Biosciences. Anti-Ly6G-PE, anti-CD11b-BV650, anti-PD-1-FITC, anti-F4/80-BV421, anti-CD115-PE, anti-Ly6C-APC/Cyanine7, and anti-PD-L1-PE-Cy7 were purchased from Biolegend. Red blood cells (RBC) were lysed using an RBC lysis buffer [(0.83% w/v ammonium chloride (Merck, cat. # 1011450500)] + 0.1% w/v potassium bicarbonate (Merck, cat. # 237205) + 100 µM EDTA (1^st^ Base, cat. # BUF-1052-pH8.0)). For surface staining, splenocytes or blood cells were washed and incubated with antibodies for 30 min on ice. The cells were then fixed and were read on a Cytek Aurora Spectral Flow Cytometer and analyzed using FlowJo software (Tree Star).

### CD8^+^ T cells restimulation assay

5-6-wk-old IFNAR^−/−^ mice were infected sc. with 10^6^ PFU of WT or T209L DENV. At day 5 p.i., the spleens were harvested and CD8^+^ T cells were isolated using MACs CD8a (Ly-2) microbeads (Miltenyi Biotech). CD8^+^ T cells (0.5 × 10^5^ or 1 × 10^5^) were co-cultured with splenic dendritic cells (isolated from WT C57BL/6J) at 1:1 ratio in the presence of 10 µg/ml of NS4B_99-107_ peptide for 20 h in an ELISPOT plate pre-coated with anti-mouse IFNγ or anti-mouse granzyme b. After 20 h, cells were discarded and biotinylated detection antibody, enzyme conjugate and chromogenic substrate solution were added sequentially. The number of spot-forming units was enumerated using an ELISPOT plate reader (Mabtech).

### Native PAGE and western blot analysis

Purified NS1 proteins (1 μg) were separated on 8% Native PAGE (ThermoFisher Scientific, cat. # EA0375BOX) and protein bands were transferred to a nitrocellulose membrane (BIO-RAD, cat. # 1704271). The nitrocellulose membranes were blocked in TBS buffer 0.1% Tween 20 containing 5% nonfat dry milk for 1 h. NS1 proteins were detected using DENV2 NS1 polyclonal antibody (1:1000 ThermoFisher Scientific) with overnight incubation at 4 °C, followed by incubation with HRP-conjugated anti-rabbit IgG antibody. Clarity™ Western ECL substrate (BIO-RAD, cat. # 1705061) was used for protein detection and exposed to film.

### Bulk and single cell RNA sequencing

In all, 5–6-week-old IFNAR^−/−^ mice were infected sc. with 10^6^ PFU of WT or T209L DENV. Blood was collected at day 3 p.i. and centrifuged at 6000 × *g* for 10 min. After RBC lysis ((0.83% w/v ammonium chloride (Merck, cat. # 1011450500) + 0.1% w/v potassium bicarbonate (Merck, cat. # 237205) + 100 µM EDTA (1st Base, cat. # BUF-1052-pH8.0)), cells were washed twice in RPMI (Gibco) supplemented with 10% FBS (Gibco). RNA was extracted from the cell pellet by adding 1 mL of TRIzol (Invitrogen, cat. # 15596026) and incubated for 5 min at room temperature. Subsequently, 200 μL chloroform was added to each sample and mixed vigorously for 15 s and incubated for 3 min. Mixture was then centrifuged at 12,000 × *g* at 4 °C for 15 min to collect the aqueous phase of the mixture, which was collected into a new clean tube. 1 mL of 100% isopropanol was then added to the aqueous phase and incubated for 10 min at room temperature. Further RNA extraction was then performed using RNeasy mini kit (Qiagen) as per manufacturer’s protocol.

For scRNAseq analysis, red blood cells were completely lysed from mouse blood samples using BD Pharm Lyse™ Lysing Buffer (BD). Dead cells were removed with dead Cell Removal kit (Miltenyi Biotec). All single cell suspensions achieved >80% viability before fixation. Samples with approximately 700,000-3 million cells were fixed overnight at 4 °C and stored at −80 °C, according to the 10x Genomics Demonstrated Protocol CG000478. Fixed cells (300,000–2 million cells) were hybridised overnight with both mouse whole transcriptome amplification (WTA) probes and spike-in custom probe pool, each probe at 40 nM (LHS and RHS combined). Single cell RNA sequencing libraries were then generated, with a targeted cell recovery of 10,000 cells, using Chromium Fixed RNA Profiling Kit (10x Genomics). Libraries were sequenced on the Illumina NovaSeq 6000 instrument using 150 paired-end reads.

For analysis of bulk RNASeq results, raw counts were analysed using the Partek® Genomics Suite®. Differentially expressed genes (DEGs) were identified by comparing gene expression between T209L NS1 mutant and WT, based on fold-change >2.0 and false discovery rate adjusted *P* value < 0.05 using the Benjamini–Hochberg step-up FDR-controlling procedure. For pathway analysis, the upregulated and downregulated DEGs were analysed against the Gene Ontology Biological Process (GOBP) and Reactome databases using the EnrichR tool (Kuleshov et al, 2016). The DEGs enriched in the respective enriched pathways, namely inflammatory response and neutrophil degranulation pathways were also extracted, normalised using Z-score transformation and the relative expression was plotted on clustergrams using on numpy, pandas and seaborn packages in Python.

For analysis of scRNASeq results, raw sequencing data was processed using the Cell Ranger pipeline (10x Genomics). The filtered gene-barcode matrix of UMI counts was then analyzed with Seurat (v5.0.3) for quality control, normalization, dimensional reduction, integration, clustering, and visualization V5 (Stuart et al, 2019). Quality control criteria were: (1) total UMI count between 500 and 30,000, (2) minimal number of detected genes >300, (3) mitochondrial gene percentage <20% and (4) number of cells expressing a gene >10. The count matrix was log-normalized, and the top 2000 most variable genes were identified for dimensional reduction. The matrix was then scaled, and the top 53 PCA dimensions were used for the uniform manifold approximation and projection (UMAP). The top 53 PCs were chosen based on the Jackstraw and Elbow plot method. The data were then integrated using the canonical correlation analysis (CCA) in Seurat (v5.0.3). Settings were kept at default unless specified otherwise. Cell clusters were identified by the shared nearest neighbor (SNN) method using the Louvain algorithm with a 2.3 resolution. Annotations were based on the markers in Fig. EV3. Markers were common cell type markers derived from published single-cell analysis and Celltypist (Xu et al, 2023).

### Glycoproteomics

Purified NS1 protein samples were treated using the single-pot, solid-phase-enhanced sample preparation (SP3) protocol (Hughes et al, 2019). In brief, 30 µg of NS1 protein were denatured and reduced using 8 M urea/50 mM Ambic and 10 mM DTT at 60 °C for 30 min followed by alkylation with IDA in the dark at room temperature for 30 min. The alkylation reaction was subsequently quenched by the addition of 15 µL of 10 mM DTT and incubation at room temperature for 15 min.

Sera-Mag carboxylated speedbeads (Cytiva, Marlborough, MA) were added to the samples in 1:10 (wt/wt) protein/beads ratio. Acetonitrile was added to the suspension to a final concentration of 70% ACN (v/v) and the final mixture was then incubated at 24 °C with mixing at 1,200 rpm for 10 min. After 10 min, the magnetic beads were immobilized using a magnetic stand and the supernatant was removed. The magnetic beads were rinsed with 80% ACN and the beads were immobilized again to discard the washing. This was repeated another two times before the beads were reconstituted in 50 mM Ambic. Sequencing grade trypsin/lysC (Promega, Madison, WI) was added in 1:20 (wt/wt) enzyme/protein ratio to the beads and incubated at 37 °C for 16 h.

After 16 h, the beads were centrifuged for 1 min at 20,000 × *g*, immobilized on the magnetic stand and the supernatant collected into a clean tube. The supernatant was dried down using a vacuum centrifuge and stored at −20 °C until the next step of analysis.

For glycopeptide enrichment, 30 μg of the sample after SP3 treatment were reconstituted in 30 µL of LCMS grade water and 10 µL of Sera-Mag carboxylated speedbeads were added to the sample. Acetonitrile was then added to the mixture to a final concentration of 80% (v/v). The suspension was incubated at 24 °C with mixing at 1200 rpm for 15 min. After 15 min, the magnetic beads were immobilized using a magnetic stand and the supernatant was removed. The magnetic beads were rinsed thrice with 80% ACN. LCMS grade water was added to the beads and incubated at 24 °C with mixing at 1,200 rpm for 15 min to elute the bound glycopeptides. The eluted glycopeptides were collected in a clean tube and dried down. The dried sample was reconstituted in LCMS water and injected into the LCMS for analysis.

In all, 18 μg of the glycopeptide enriched sample were injected onto a Dionex Ultimate RSLC 3000 nano LC system (Thermo Fisher Scientific, Waltham, MA) coupled to an Orbitrap Fusion Tribrid mass spectrometry system (Thermo Fisher Scientific, Waltham, MA) with an Easy-Spray™ nano source. The samples were trapped on a C18 trap column (Acclaim PepMap100 C18, 5 µm, 300 µm × 5 mm, Thermo Fisher Scientific, Waltham, MA) for 2 min before separation on an Easy-Spray™ C18 nano column (75 µm × 150 mm, 2 µm, Thermo Fisher Scientific, Waltham, MA) with a 107 min gradient at a flowrate of 0.3 µL/min. Solvent A was 0.1% formic acid in water and solvent B was 0.1% formic acid in ACN.

Mass spectrometry data was acquired in the positive ion data dependent acquisition (DDA) mode. Full scan MS spectra data were acquired in the mass range of *m/z* 500 to 2000, at an ionization source voltage of 2.2 kV, using the Orbitrap detector with resolution set to 120 K, normalized AGC target at 100%, maximum injection time of 50 ms. MS/MS data was acquired using a top speed mode of 5 s in the Orbitrap detector at 30 K resolution, with an isolation window of 1.6 *m/z*, stepped HCD collision energy of 20%, 30% and 45%, normalized AGC target of 200% and maximum injection time of 100 ms. The criteria for MS/MS to be triggered for the precursor ions were minimum intensity of 5e4, charge states of 2 to 6. Dynamic exclusion was set at a duration of 15 s within a mass tolerance of 15 ppm.

Data analysis was performed using Thermo Proteome Discoverer (v2.2.0.388, Thermo Fisher Scientific, Waltham, MA) integrated with Byonic™ (v3.9.6, Protein Metrics, Cupertino, CA) node. The raw files were first matched against Swiss-Prot proteome database with the targeted NS1 protein sequence included. Software parameters were set as follows: missed cleavages set to two, digestion enzyme set to trypsin (full), precursor mass tolerance at 15 ppm, fragment mass tolerance at 20 ppm, fragmentation type to HCD, with modifications of methionine oxidations and deamidation of asparagine and glutamine set as dynamic and carbamidomethylations of cysteine set as static. N-glycosylation modifications were searched against the N-glycans mammalian database by Byonic™. The false discovery rate (FDR) was set to 1%. A focused database with decoys was created from the initial analysis and the focused database was used subsequently for all samples analysed. For samples that have mutations at a particular amino acid, the amino acid mutation was included as part of the modification parameters and set to dynamic.

Quantification of glycopeptides was based on peak area performed using the Minora feature detector and precursor ions quantifier nodes in Thermo Proteome Discoverer.

### Lipidomics analysis

Purified NS1 protein samples (50 µL) were mixed with 450 µL 1-butanol/methanol (BuMe, 1:1 v/v) containing lipid internal standards and vortexed for 30 s, followed by 30 min sonication at 20 °C. The samples were centrifuged at 14,000 × *g* for 10 min at 10 °C. A volume of 400 µL of the supernatant was carefully transferred into vials and dried down completely using a vacuum concentrator system, then resuspended in 100 µL 1-butanol/methanol (BuMe, 1:1 v/v) before MS analysis.

The analysis was carried out on an Agilent 6495 QQQ and Infinity-II LC-MS system, using a Zorbax RRHD Eclipse Plus C18, 95 Å (2.1 × 50 mm, 1.8 µm) column. The mobile phases consisted of (A) 10 mmol/L ammonium formate and formic acid (0.1%) in water/acetonitrile (60:40, v/v) and (B) 10 mmol/L ammonium formate and formic acid (0.1%) in 2-propanol/acetonitrile (90:10, v/v). Using a flow rate of 0.4 mL/min, the gradient started from 20% B to 60% B in 2 min, 60 to 100% B in 10 min, where it was maintained for 2 min, then the column re-equilibrated at 20% B for 1.8 min prior to the next injection. The injection volume was 2 µL. Autosampler and column thermostat temperature were at 15 °C and 45 °C, respectively. Total method run time was 15.8 min.

The mass spectrometer conditions were as follows: Capillary voltage 3000 V, drying and sheet gas temperatures were 250 °C, drying and sheet gas flow rates were 14 L/min and 17 L/min, respectively and the nebulizer nitrogen gas flow was set to 35 psi. A semi-targeted analysis, covering the most abundant lipid classes in mammals, was performed in dynamic MRM positive ion mode, using “unit” resolution (0.7 amu) for Q1 and Q3 isolation width. Chromatographic peaks were annotated based on retention time and specific MRM transitions using Agilent MassHunter Quantitative Analysis software (version B.12.0). Internal standards were used to normalize the raw peak areas in the corresponding lipid class and the final lipid concentrations were expressed as nanomole of lipid per g of protein (NS1 protein in the samples).

### Molecular dynamics simulations

It was previously estimated that ~70 lipid molecules can be extracted from NS1 hexamer (Gutsche et al, 2011). D2Y98P strain experimental lipidomics data were used and only lipids with their count greater than 1% of total lipids were considered. This corresponded to PC:PE:PI:SM with 11:3:83:3 ratio, respectively. In total 8 POPC, 2 POPE, 58 SLPI and 2 PSM lipid were randomly placed in a cubic box of 15x15x15 nm (Jo et al, 2008). Approximately 100,000 TIP3P water molecules were added to the box and 150 mM NaCl salt whilst neutralizing the overall system charge. Energy minimization was performed using the steepest descent (SD) minimization algorithm with a 0.01 nm energy step size. Unrestrained production run was run for 500 ns in the NPT ensemble. The preequilibrated micelle was subsequently used for simulations with NS1 hexamer. The experimental structure (PDB: 4O6B) (Akey et al, 2014) of the NS1 hexamer dengue serotype 2 was used. In silico mutations to experimentally studied strain D2Y98P were performed using CHARMM-GUI (Jo et al, 2008). Protein charges were assigned according to neutral pH with charged termini. The wild-type (WT) NS1 hexamer N-linked glycan composition at site N130 corresponded to Hex:3 HexNAc:7, while N207 site corresponded to Hex:6 HexNAc:3 according to experimental data (Appendix Fig. S1). The preequilibrated micelle was placed in the center of the hexamer hydrophobic β-roll domains. All lipids overlapping with protein based on the 0.3 nm cutoff distance were moved away from the hydrophobic domain (residues 1-29). The energy minimization in vacuum was performed using SD algorithm. The WT construct was placed in a dodecahedron box with ~17 nm box edge. Approximately 90,000 TIP3P water molecules were added to the box and 150 mM NaCl salt whilst neutralizing the overall system charge. Energy minimization was performed using the steepest descent minimization algorithm with a 0.01 nm step size. The system was equilibrated in the NPT ensemble for 200 ns with position restraints on protein alpha carbons and a force constant of 1000 kJ mol^−1^ nm^−2^. The equilibrated i) WT-lipid complex was used to generate mutation T209L, which resulted in loss of glycans at position N207. Both systems were subjected to a production run of 500 ns in the NPT ensemble which involved weak position restraints on protein alpha carbons with a force constant of 100 kJ mol^−1^ nm^−2^. All simulations were performed using the GROMACS 2018.3 simulation package (Van Der Spoel et al, 2005) utilizing the CHARMM36m (Huang et al, 2017) force field with the TIP3P water model (Jorgensen et al, 1983). A temperature of 310 K was maintained using the velocity rescaling thermostat with an additional stochastic term using a time constant of 1 ps. Pressure was maintained semi-isotropically at 1 atm using the Parrinello-Rahman barostat (Parrinello and Rahman, 1981) and a time constant of 5 ps. All bonds which involved hydrogens were constrained using the LINCS algorithm. Equations of motion were integrated using the leap-frog algorithm with a time step of 2 fs. Long-range electrostatic interactions were described using the particle mesh Ewald method (Essmann et al, 1995). The short-range electrostatics reals pace cut-off was 1.2 nm and the short-range van der Waals cut-off was also 1.2 nm. Periodic boundaries conditions were applied in all directions. Simulations were performed on: i) an in-house Linux cluster composed of 8 nodes containing 2 GPUs (Nvidia GeForce RTX 2080 Ti) and 24 CPUs (Intel® Xeon® Gold 5118 CPU @ 2.3 GHz) each as well as on ii) National Supercomputing Center (https://www.nscc.sg) using 2 nodes containing 128 cores and 256 logical cores (AMD EPYC™ 7713 @ 2.0 GHz) each. All simulations snapshots were generated using VMD (Humphrey et al, 1996). The glycan–lipid contacts were calculated based on the 0.3 nm cutoff distance and were averaged over last 100 ns of trajectory. The buried solvent accessible surface area (SASA) between NS1:glycans and lipid cargo was calculated as a sum of NS1:glycans and lipids before subtracting (NS1:glycan)–lipid SASA.

### Statistical analyses

Experimental groups were not blinded. Data analyses were performed using Graphpad Prism 9.0. Statistical comparison was conducted using nonparametric Mann–Whitney *U* test or two-way ANOVA and Sidak’s multiple comparison test. Comparison of survival rates was performed using log-rank (Mantel–Cox) test. Differences were considered significant at *P* < 0.05.

### Ethics statement

All animal experiments were carried out in accordance with the guidelines of the National Advisory Committee for Laboratory Animal Research. Animal facilities are licensed by the regulatory body Agri-Food and Veterinary Authority of Singapore. The described animal experiments were approved by the Institutional Animal Care and Use Committee from National University of Singapore (NUS) under protocol numbers R16-0422 and R18-1400. Mice were housed in AAALAC-accredited ABSL2 facility, and husbandry was performed according to the institutional guidelines. Males and females were used in the experiments, and were randomly allocated to the various groups, ensuring comparable gender ratios across the groups. Genetic modifications of DENV were approved by the Genetic Modification Advisory Committee (GMAC) and the Institutional Biosafety Committee (IBC).

## Supplementary information

Appendix

Peer Review File

Source data Fig. 1

Source data Fig. 2

Source data Fig. 3

Source data Fig. 4

Source data Fig. 5

Source data Fig. 6

Source data Fig. 7

Expanded View Figures

## Data Availability

(1) RNAseq data repository: The RNAseq data have been deposited to the ArrayExpress via the Annotare partner repository with the dataset identifier E-MTAB-15296. Data for RNAseq on ArrayExpress can be accessed through: https://ddec1-0-en-ctp.trendmicro.com:443/wis/clicktime/v1/query?url=https%3a%2f%2fwww.ebi.ac.uk%2fbiostudies%2farrayexpress%2fstudies%2fE%2dMTAB%2d15296%3fkey%3d8426c0b3%2d277c%2d4e55%2da011%2dc3aaf72588cf&umid=8b2c7cd6-6bf2-4092-a8b4-0c53b723ce7b&rct=1751449092&auth=3eee6d57317a38631073652579c10dc620ca2b41-a8cfa97cb6b3e10c8de2cb13c388c99980cf4c89. (2) Glycoproteomics data repository: The mass spectrometry proteomics data have been deposited to the ProteomeXchange Consortium via the PRIDE partner repository with the dataset identifier PXD065422. Reviewer access details: Log in to the PRIDE website (https://www.ebi.ac.uk/pride/login) using the following details: Project accession: PXD065422. Token: aQtNat2Gq6fo. Alternatively, reviewer can access the dataset by logging in to the PRIDE website using the following account details: Username: reviewer_pxd065422@ebi.ac.uk. Password: 24c1qpErGjv4. The source data of this paper are collected in the following database record: biostudies:S-SCDT-10_1038-S44321-025-00311-6.
