# Supplementary material for: Glycans on non-structural protein 1 prevent premature T-cell mediated dengue virus clearance
Source: EMBO Mol Med. 2025 Sep 17;17(11):2995–3020. doi: 10.1038/s44321-025-00311-6 (PMC12603335; doi:10.1038/s44321-025-00311-6)
Supplement: Supplementary file 1 — Appendix [file 44321_2025_311_MOESM1_ESM.pdf]

Appendix

	Title	Page
**Appendix Table S1**	De-glycosylated NS1 mutants generated by site-directed mutagenesis.	2
**Appendix Table S2**	Primer pairs used for virus genome amplification and site-directed mutagenesis.	3
**Appendix Figure S1**	Glycan profiles of WT and T209L sNS1.	4
**Appendix Figure S2**	Co-infection experiment (10exp6 PFU dose).	5
**Appendix Figure S3**	$CD8^+$ T-cell depletion in WT DENV-infected mice.	6
**Appendix Figure S4**	Vascular leakage in mice infected with WT or T209L DENV.	7

Appendix Table S1. De-glycosylated NS1 mutants generated by site-directed mutagenesis. Mutation stability was assessed after 3 consecutive passages in C6/36 cell line.

Mutants	Amino acid substitution	Change in amino acid chemical property	Features
N130Q	Asn to Glu	No change	Stable after 3 passages
N207H	Asn to His	Polar to basic	Reversion after 3 passages
N207Q	Asn to Glu	No change	Reversion after 3 passages
T209A	Thr to Ala	Polar to non-polar	Reversion after 3 passages
T209L	Thr to Leu	Polar to non-polar	Stable after 3 passages
T209V	Thr to Val	Polar to non-polar	Reversion after 3 passages

Appendix Table S2. Primer pairs used for virus genome amplification and site-directed mutagenesis.

Primer Name	Primer Sequence (5' to 3')
D2Y98P-F1-FP	CTGGTTTAGTGAACCGTCAGAGTAGTTAGTCTACGTGGAC
D2Y98P-F1-RP	CTCACAACGCAACCACTATCGGCCTGCACCATAACTCC
D2Y98P-F2-FP	TGGGAGTTATGGTGCAGGCCGATAGTGGTTGCGTTGTG
D2Y98P-F2-RP	ATTGCTGGAAGGTATCTCTTTGTTTTTCCTGCTCCTGG
D2Y98P-F3-FP	ACCCAGGAGCAGGAAAAACAAAGAGATACCTTCCAGCAATAGTCAGAGAAG
D2Y98P-F3-RP	TTTGAAGACGCACCAGATTCCAACCATATGTTGACATGG
D2Y98P-F4-FP	CCCATGTCAACATATGGTTGGAATCTGGTGCGTCTTCAAAG
D2Y98P-F4-RP	TGGAGATGCCATGCCGACCCAGAACCTGTTGATTCAAC
Vector (CMV, HDV ribozyme and SV40 PA) FP	CTGTTGAATCAACAGGTTCTGGGTCGGCATGGCATCTC
Vector (CMV, HDV ribozyme and SV40 PA) RP	GTCCACGTAGACTAACTACTCTGACGGTTCACTAAACCAGC
130 N-Q FP	CTCTCCACAGAGCTTCATCAACACACCTTTCTCATTGA
130 N-Q RP	CAATGAGAAAGGTGTGTTGATGAAGCTCTGTGGAGAGCAT
209 T-L FP	CTCAATGACCTATGGAAGATTGAGAAAGCCTC
209 T-L RP	GAGGCTTTCTCAATCTTCCATAGGTCATTGAG

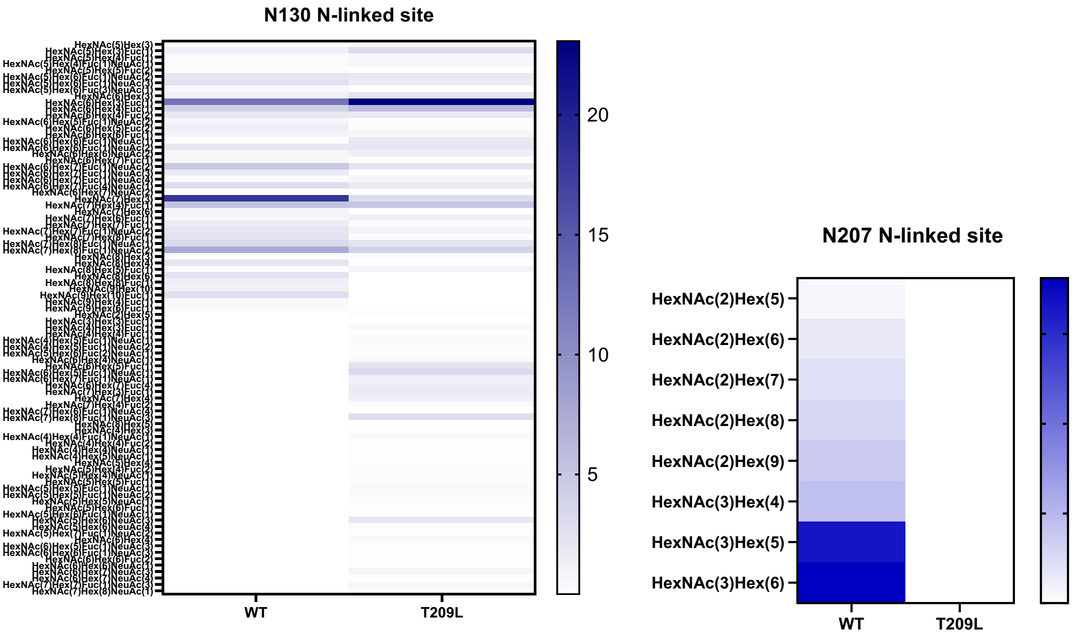

Appendix Figure S1. Glycan profiles of WT and T209L sNS1.

Identity and relative abundance of glycan species present at both N-glycosylation sites on purified WT and T209L sNS1 proteins, as determined by glycomics and glycoproteomics. Data shown are from one biological repeat.

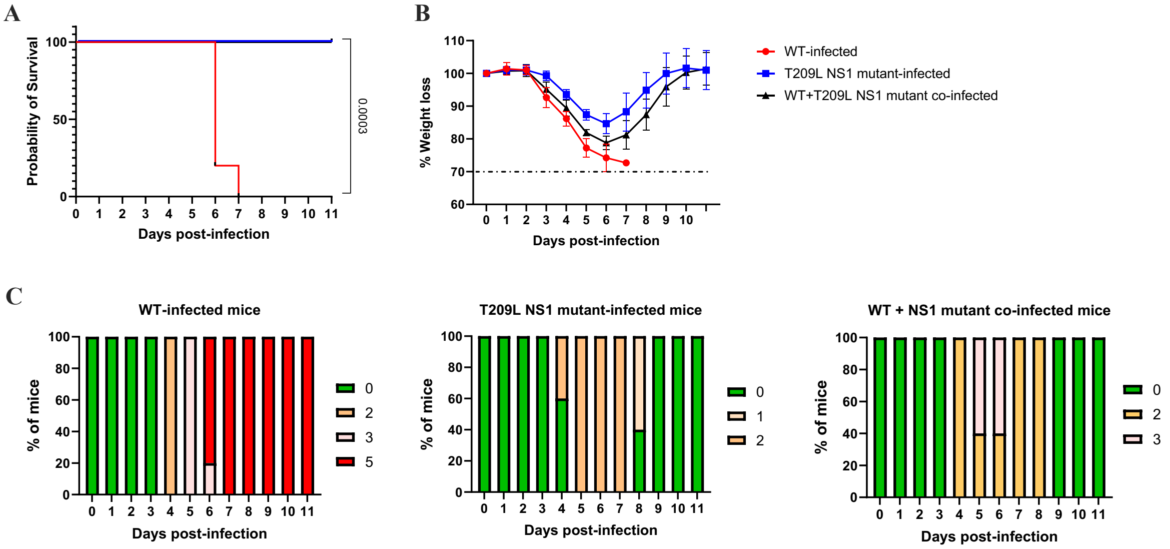

Appendix Figure S2. Co-infection experiment (10exp6 PFU dose).

IFNAR$^{-/-}$ mice were sc. infected with either WT or T209L mutant (10^6 PFU per mouse) or were co-infected with WT and T209L NS1 mutant viruses ($1x10^6$ PFU of each virus). (A) Survival rate (n=5), (B) Body weight profile (n=5). (C) Clinical scores as described in the legend of Fig. 1 (n=5). 0 – healthy, 1 – ruffled fur, 2 – hunched back, 3 – lethargy, 4 – limb paralysis, 5 – mice displaying 30% weight loss (euthanasia). Data shown are from one biological repeat. All graphs were expressed as mean ± SD. Data analysis were performed using Log-rank (Mantel-Cox) test (A).

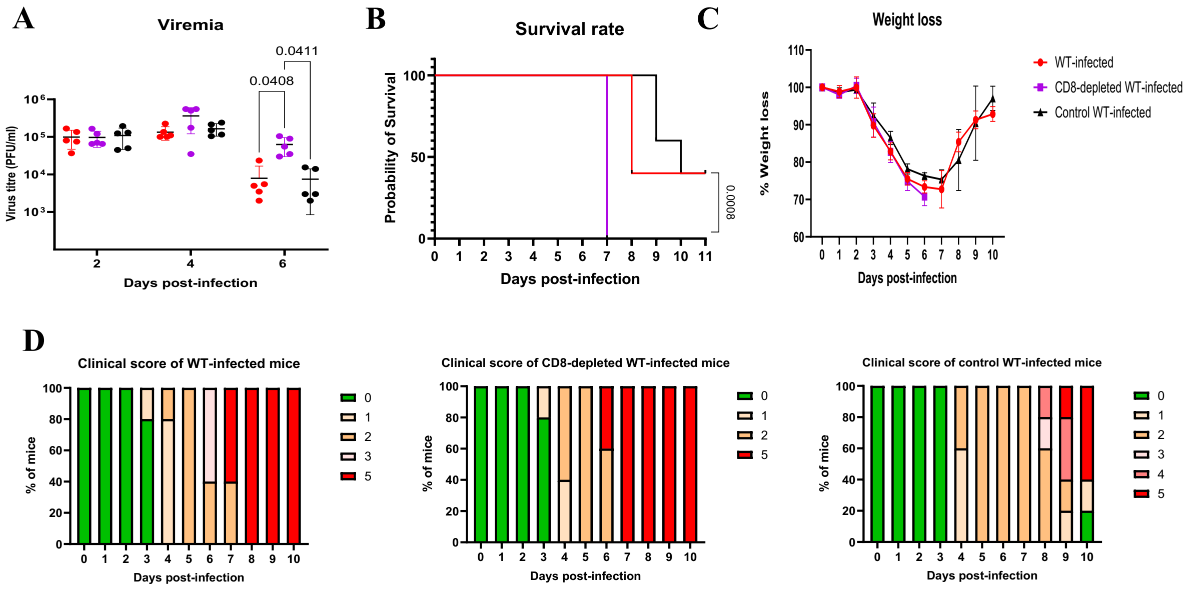

Appendix Figure S3. $CD8^{+}$ T-cell depletion in WT DENV-infected mice.

(A) Viremia titers were measured by plaque assay (n=5). (B) Survival rate (n=5). (C) Body weight profile (n=5). (D) Clinical scores as described in the legend of Fig. 1 (n=5). Data shown are from one biological repeat. All graphs were expressed as mean ± SD. Data analysis were performed using two-way ANOVA Tukey's multiple comparisons test (A) and Log-rank (Mantel-Cox) test (B).

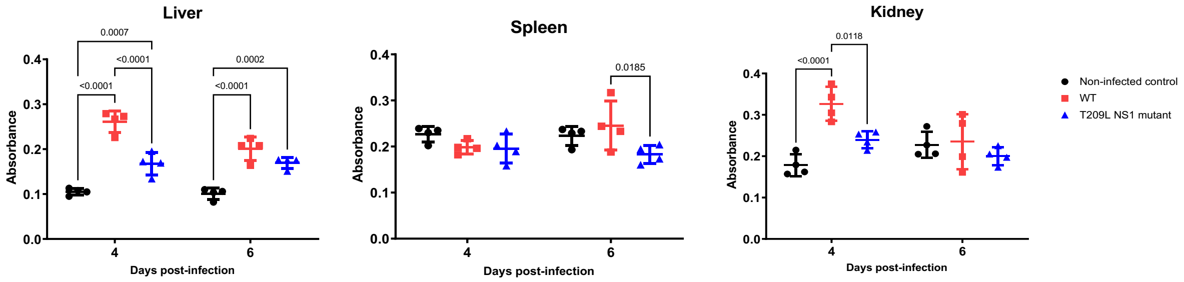

Appendix Figure S4. Vascular leakage in mice infected with WT or T209L DENV.

Vascular leakage was determined by Evans Blue assay in mice infected with WT or T209L DENV at day 4 and 6 p.i. (n=4, one biological repeat). All graphs were expressed as mean ± SD. Data analysis were performed using two-way ANOVA Tukey's multiple comparisons test.
