# Supplementary figures and images for: Glycans on non-structural protein 1 prevent premature T-cell mediated dengue virus clearance
Source: EMBO Mol Med. 2025 Sep 17;17(11):2995–3020. doi: 10.1038/s44321-025-00311-6 (PMC12603335; doi:10.1038/s44321-025-00311-6)

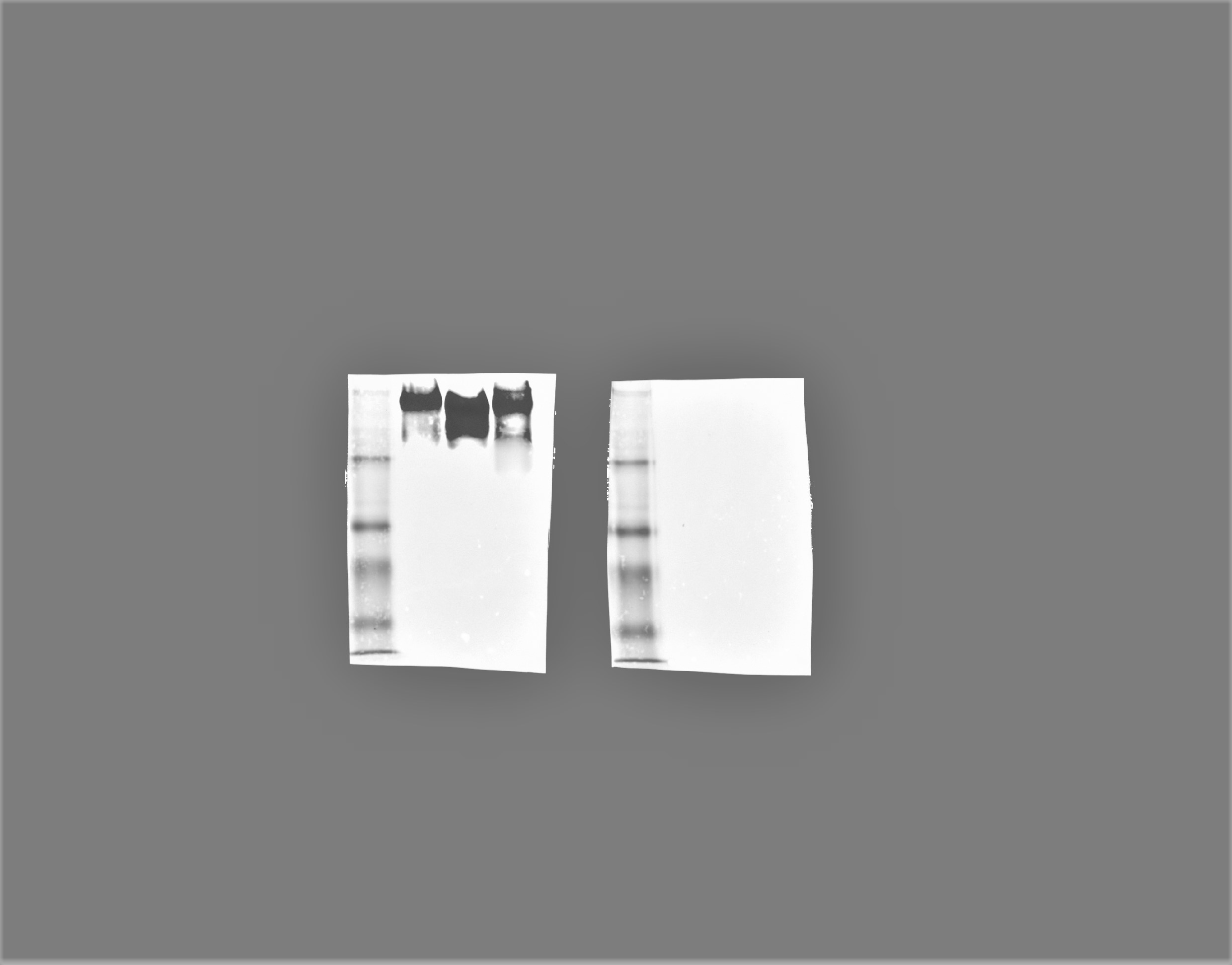

Supplement: Supplementary file 4 — Source data Fig. 2 [file 44321_2025_311_MOESM4_ESM.zip › Figure 2/Figure 2B Hexameric NS1 WB images.jpg]
