# Supplementary material for: Glycans on non-structural protein 1 prevent premature T-cell mediated dengue virus clearance
Source: EMBO Mol Med. 2025 Sep 17;17(11):2995–3020. doi: 10.1038/s44321-025-00311-6 (PMC12603335; doi:10.1038/s44321-025-00311-6)
Supplement: Supplementary file 10 — Expanded View Figures [file 44321_2025_311_MOESM10_ESM.pdf]

Expanded View Figures

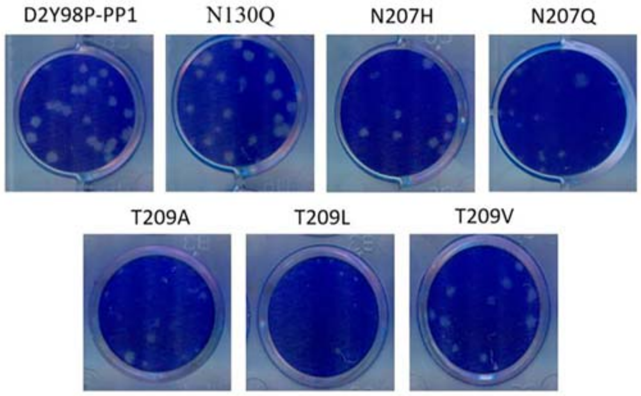

Figure EV1. Plaque morphology of WT and de-glycosylated NS1 DENV mutants.

The viruses were amplified in C6/36 cells and plaqued on BHK-21 cells. Data shown are representative of at least 2 independent biological repeats.

 © The Author(s)

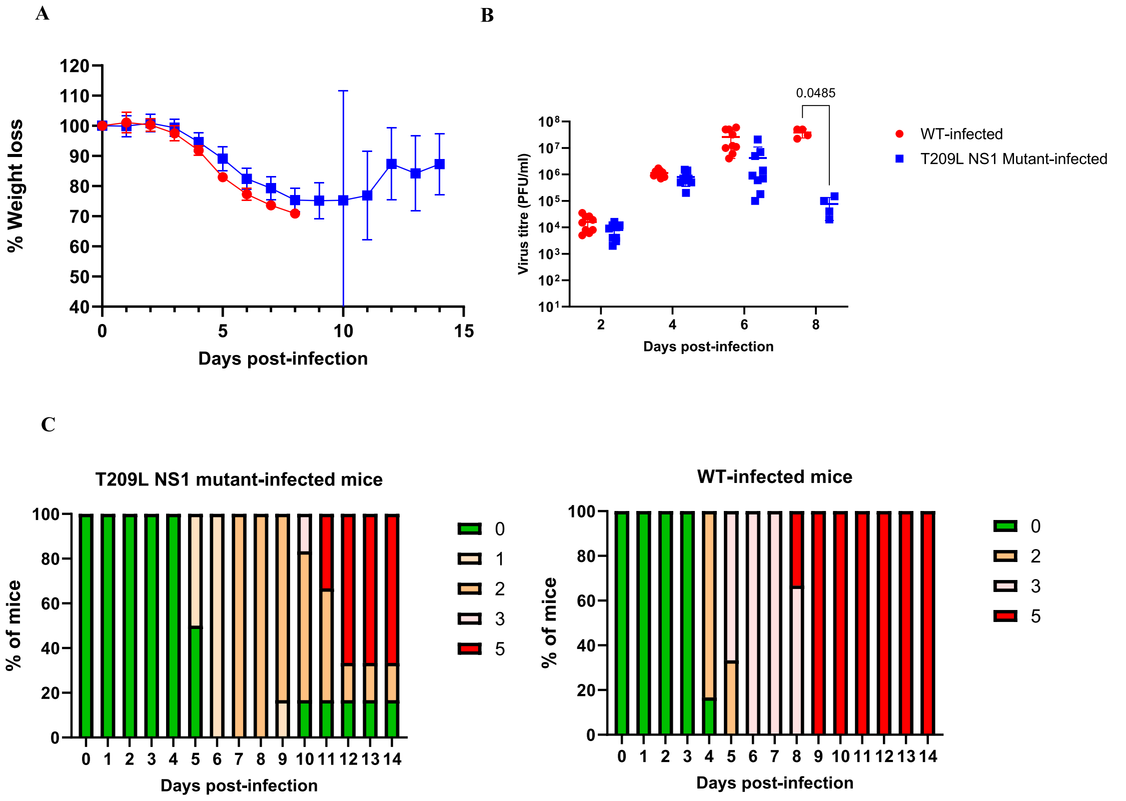

Figure EV2. Infection profile of WT and T209L DENV in IFNAR-muMT mice.

(**A**) Body weight profile ($n = 9$). (**B**) Viremia titers were determined by plaque assay ($n = 4$–9). (**C**) Clinical scores as described in the legend of Fig. 1 ($n = 9$). Data shown are a combination of 2 independent repeats. All graphs were expressed as mean ± SD. Data analysis were performed using two-way ANOVA Sidak's multiple comparisons test (**B**).

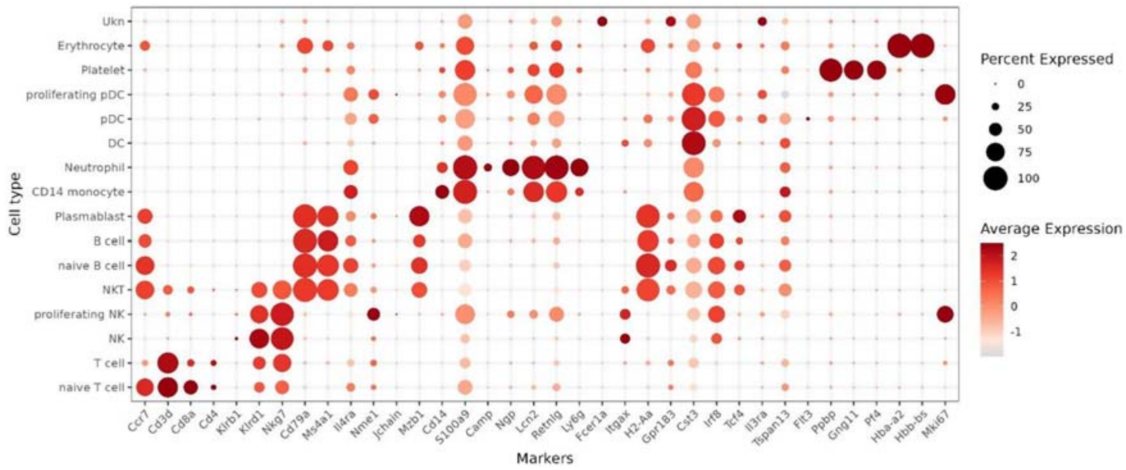

Figure EV3. Relative abundance of markers of immune cell clusters used for single cell RNAseq data analysis.

 © The Author(s)

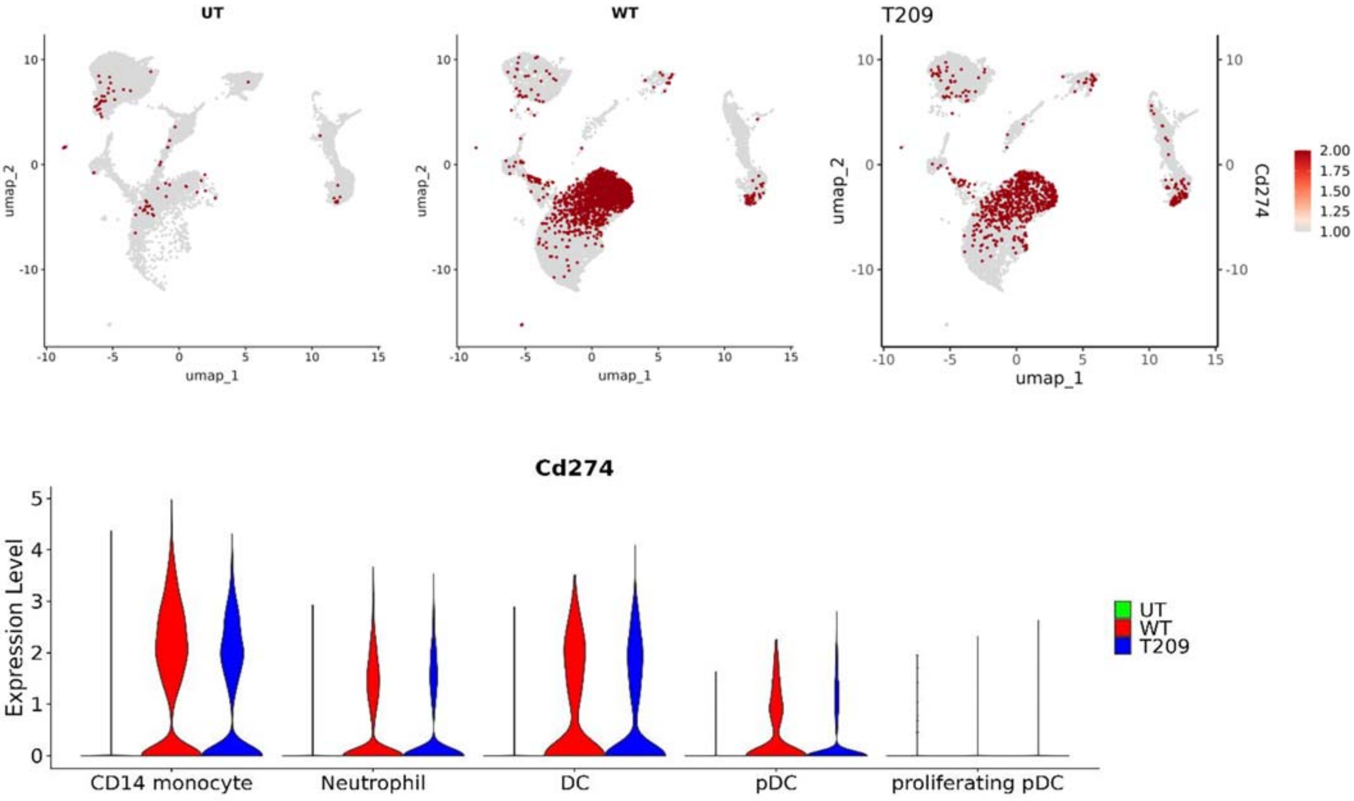

Figure EV4. PD-L1 (CD274) gene expression in neutrophils, monocytes and dendritic cells in DENV-infected mice.

The expression of PD-L1 gene in monocytes, neutrophils and dendritic cell subsets in WT- and T209L DENV-infected IFNAR$^{-/-}$ mice was determined by single cell RNAseq.

© The Author(s)

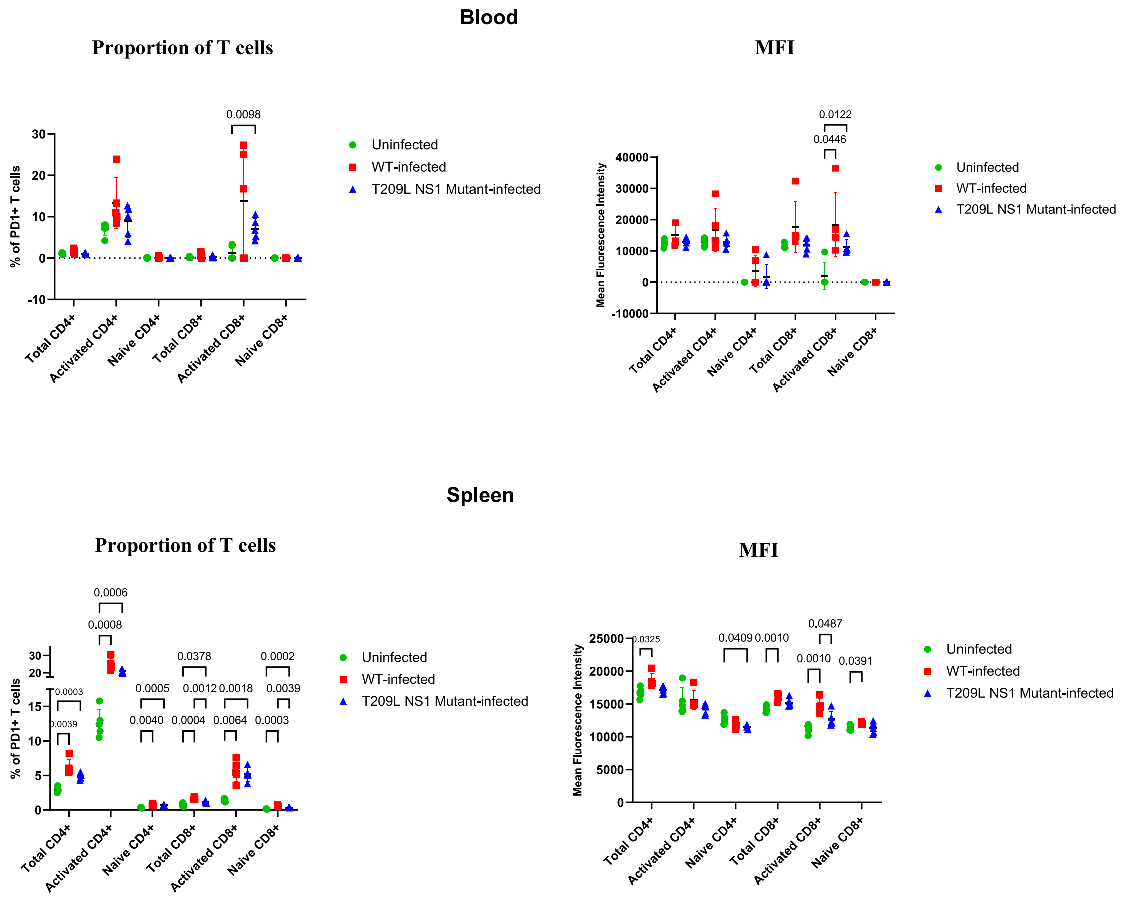

Figure EV5. PD-1 expression on T cells.

Flow cytometry analysis of circulating and splenic T cells harvested at day 3 p.i. from WT- or T209L NS1 DENV-infected IFNAR$^{-/-}$ mice ($n = 5$). Percentages of PD-1^{+} T cells and mean fluorescence intensity (MFI) of PD-1 expression on T cells. Data shown are from one biological repeat. All graphs were expressed as mean ± SD. Data analysis were performed using two-way ANOVA Tukey's multiple comparisons test.

© The Author(s)
