# Supplementary material for: Glycans on non-structural protein 1 prevent premature T-cell mediated dengue virus clearance
Source: EMBO Mol Med. 2025 Sep 17;17(11):2995–3020. doi: 10.1038/s44321-025-00311-6 (PMC12603335; doi:10.1038/s44321-025-00311-6)
Supplement: Supplementary file 2 — Peer Review File [file 44321_2025_311_MOESM2_ESM.pdf]

EMBO Molecular Medicine

Glycans on Non-Structural Protein 1 Prevent Premature T-cell Mediated Dengue Virus Clearance

Fakhriedzwan Idris, Justin Ooi, Donald Ting, Eunice Tan, Corrine Wan, Peter Benke, Jan Marzinek, Jack Copping, QinHui Li, LuYi Ng, Sheau Yng Lim, Ian Walsh, Jane Allison, Peter Bond, Federico Torta, Terry Nguyen-Khuong, Kuan Rong Chan, and Sylvie Alonso

Corresponding author: Sylvie Alonso (micas@nus.edu.sg)

Review Timeline:

Submission Date:	28th Feb 25
Editorial Decision:	24th Mar 25
Revision Received:	14th Jul 25
Editorial Decision:	25th Jul 25
Revision Received:	18th Aug 25
Editorial Decision:	26th Aug 25
Revision Received:	3rd Sep 25
Accepted:	3rd Sep 25

Editor: Zeljko Durdevic

Transaction Report:

(Note: With the exception of the correction of typographical or spelling errors that could be a source of ambiguity, letters and reports are not edited. Depending on transfer agreements, referee reports obtained elsewhere may or may not be included in this compilation. Referee reports are anonymous unless the Referee chooses to sign their reports.)

24th Mar 2025

Dear Prof. Alonso,

Thank you for the submission of your manuscript to EMBO Molecular Medicine. We have now received feedback from the two reviewers who agreed to evaluate your manuscript. Both referees recognize interest of the study but also raise serious concerns that should be addressed in a major revision. Considering that the revision will require extensive experimentation we think six months rather than three months would be more appropriate to provide the complete revision. If you would like to discuss further the points raised by the referees, I am available to do so via email or video. Let me know if you are interested in this option.

Further consideration of a revision that addresses reviewers' concerns in full will entail a second round of review. EMBO Molecular Medicine encourages a single round of revision only and therefore, acceptance or rejection of the manuscript will depend on the completeness of your responses included in the next, final version of the manuscript. For this reason, and to save you from any frustrations in the end, I would strongly advise against returning an incomplete revision.

We would welcome the submission of a revised version within six months for further consideration. Please let us know if you require longer to complete the revision.

I look forward to receiving your revised manuscript.

Yours sincerely,

Zeljko Durdevic

Zeljko Durdevic
Editor
EMBO Molecular Medicine

*****

When submitting your revised manuscript, please carefully review the instructions that follow below. We perform an initial quality control of all revised manuscripts before re-review; failure to include requested items will delay the evaluation of your revision.

We require:

1) A .docx formatted version of the manuscript text (including legends for main figures, EV figures and tables). Please make sure that the changes are highlighted to be clearly visible.

2) Individual production quality figure files as .eps, .tif, .jpg (one file per figure). For guidance, download the 'Figure Guide PDF': (https://www.embopress.org/page/journal/17574684/authorguide#figureformat).

3) A .docx formatted letter INCLUDING the reviewers' reports and your detailed point-by-point responses to their comments. As part of the EMBO Press transparent editorial process, the point-by-point response is part of the Review Process File (RPF), which will be published alongside your paper.

4) A complete author checklist, which you can download from our author guidelines (https://www.embopress.org/page/journal/17574684/authorguide#submissionofrevisions). Please insert information in the checklist that is also reflected in the manuscript. The completed author checklist will also be part of the RPF.

5) Please note that all corresponding authors are required to supply an ORCID ID for their name upon submission of a revised manuscript.

6) It is mandatory to include a 'Data Availability' section after the Materials and Methods. Before submitting your revision, primary

datasets produced in this study need to be deposited in an appropriate public database, and the accession numbers and database listed under 'Data Availability'. Please remember to provide a reviewer password if the datasets are not yet public (see https://www.embopress.org/page/journal/17574684/authorguide#dataavailability).

In case you have no data that requires deposition in a public database, please state so in this section. Note that the Data Availability Section is restricted to new primary data that are part of this study.

7) For data quantification: please specify the name of the statistical test used to generate error bars and P values, the number (n) of independent experiments (specify technical or biological replicates) underlying each data point and the test used to calculate p-values in each figure legend. The figure legends should contain a basic description of n, P and the test applied. Graphs must include a description of the bars and the error bars (s.d., s.e.m.). See also 'Figure Legend' guidelines: https://www.embopress.org/page/journal/17574684/authorguide#figureformat

8) At EMBO Press we ask authors to provide source data for the main manuscript figures. Our source data coordinator will contact you to discuss which figure panels we would need source data for and will also provide you with helpful tips on how to upload and organize the files.

9) Our journal encourages inclusion of *data citations in the reference list* to directly cite datasets that were re-used and obtained from public databases. Data citations in the article text are distinct from normal bibliographical citations and should directly link to the database records from which the data can be accessed. In the main text, data citations are formatted as follows: "Data ref: Smith et al, 2001" or "Data ref: NCBI Sequence Read Archive PRJNA342805, 2017". In the Reference list, data citations must be labeled with "[DATASET]". A data reference must provide the database name, accession number/identifiers and a resolvable link to the landing page from which the data can be accessed at the end of the reference. Further instructions are available at .

10) We replaced Supplementary Information with Expanded View (EV) Figures and Tables that are collapsible/expandable online. A maximum of 5 EV Figures can be typeset. EV Figures should be cited as 'Figure EV1, Figure EV2" etc... in the text and their respective legends should be included in the main text after the legends of regular figures.

- For the figures that you do NOT wish to display as Expanded View figures, they should be bundled together with their legends in a single PDF file called *Appendix*, which should start with a short Table of Content. Appendix figures should be referred to in the main text as: "Appendix Figure S1, Appendix Figure S2" etc.

- Additional Tables/Datasets should be labeled and referred to as Table EV1, Dataset EV1, etc. Legends have to be provided in a separate tab in case of .xls files. Alternatively, the legend can be supplied as a separate text file (README) and zipped together with the Table/Dataset file.

See detailed instructions here:

.

11) The paper explained: EMBO Molecular Medicine articles are accompanied by a summary of the articles to emphasize the major findings in the paper and their medical implications for the non-specialist reader. Please provide a draft summary of your article highlighting

- the medical issue you are addressing,

- the results obtained and

- their clinical impact.

This may be edited to ensure that readers understand the significance and context of the research. Please refer to any of our published articles for an example.

12) Author contributions: You will be asked to provide CRediT (Contributor Role Taxonomy) terms in the submission system. These replace a narrative author contribution section in the manuscript.

13) A Conflict of Interest statement should be provided in the main text.

14) Every published paper now includes a 'Synopsis' to further enhance discoverability. Synopses are displayed on the journal webpage and are freely accessible to all readers. They include a short stand first (maximum of 300 characters, including space) as well as 2-5 one-sentences bullet points that summarizes the paper. Please write the bullet points to summarize the key NEW findings. They should be designed to be complementary to the abstract - i.e. not repeat the same text. We encourage inclusion

of key acronyms and quantitative information (maximum of 30 words / bullet point). Please use the passive voice. Please attach these in a separate file or send them by email, we will incorporate them accordingly.

Please also suggest a striking image or visual abstract to illustrate your article as a PNG file 550 px wide x 300-600 px high.

15) Include a Reagents and Tools Table as part of the Methods section, which can be downloaded from our author guidelines (https://www.embopress.org/page/journal/17574684/authorguide#structuredmethods)

EMBO Molecular Medicine has a "scooping protection" policy, whereby similar findings that are published by others during review or revision are not a criterion for rejection. Should you decide to submit a revised version, I do ask that you get in touch after three months if you have not completed it, to update us on the status.

***** Reviewer's comments *****

Referee #1 (Comments on Novelty/Model System for Author):

In their manuscript " Glycans on Non-Structural Protein 1 Prevent Premature T-cell 1 Mediated Dengue Virus Clearance" Idris and colleagues describe the in vivo attenuation of a DENV strain that is mutated in a specific NS1 glycosylation site. The data presented is novel and the generated hypothesis is certainly interesting to a broader audience. The strongest data presented in the manuscript are the experiments using antibody-mediated depletion of sNS1 as well as the addition sNS1 wildtype and mutant protein to demonstrate an attenuation of pathogenicity through the mutated sNS1 protein. Unfortunately, there is a major caveat in the co-infection experiments that relates to the dose of the inoculum and offers an alternative explanation. The authors have to demonstrate that this alternative explanation, i.e., that attenuation and reduced immune activation is simply due to lower inoculum size, is not true. The authors have to show that the immunological effects are indeed related to the mutated sNS1 protein and not simply reflect delayed infections kinetics due to lower inoculum of the more pathogenic wildtype strain. In addition, most data presented are from one representative experiment, it would be better to show all data.

Referee #1 (Remarks for Author):

Major Issues

1. In the co-infection experiments (Figures 4, 5, 6, and 7) the lower inoculum titer of the pathogenetic WT virus in the co-infected animals than in in the single infected animals (5 x 105 compared to 1 x 106 PFU) might explain the differences that were observed. As the WT and T209L DENV display differences in virulence (smaller plaques, lower titers in the blood and organs), the results might just reflect that the T209L mutant is less fit than the WT virus. Administering lower doses of the WT strain in co-infections might lead to differences in infection kinetics that change the immune response in mice. If there is a dominant effect of T209L mutant the same amount of WT as in the single-infected control should be administered. It would be best, however, to repeat the experiments with administered mutant sNS1 to clearly link the immune effects to the mutated sNS1 as proposed by the authors.
2. The plaque assays in the Supplement indicates different fitness levels of WT and T209L DENV strains even though the titers in vitro are similar. In addition, in Vero cells the infection kinetics seem to be a bit slower for the mutant. Even small changes in infection kinetics in vitro might account for the differences in infection kinetics and pathogenesis in vivo. Triplicate datapoints in cell culture have to be replaced by independent experiments to allow for definite conclusions of in vitro fitness (see also below).
3. Page 7, lane 26: lower titers are first seen at day 3 (not significant) and are significantly lower at day 4, 5, and 6. This is important as the follow up experiments were performed at day 3 when small changes in titers in blood and significant changes in the organs are seen.
4. The N130Q DENV mutant is attenuated as well (viral titers in organs), therefore the statement that lower sNS1 levels do not contribute to attenuation is misleading.
5. Figure 4: Detailed characterization of the sNS1 protein that was administered in the mouse experiments is missing, or is that the same preparation that was used in Figure 2?
6. Figure 2C: The data presented indicate that there are less lipids bound to the T209L sNS1 even though the authors state otherwise in their discussion. In many instances the difference in lipid species levels is not statistically significant, but there is the tendency of lower lipid levels bound to the mutated sNS1. Analysis at lipid class level could reveal if there are relevant changes. In addition, the number of independent experiments is missing, if the data represents replicates and not independent experiments the statistical analysis is problematic.
7. Page 9, lane 23 and Figure 4C: Contrary to what is stated, viremia titers are not lowered by administration of T209L mutant sNS1 to WT infected animals.
8. Figure 5 C-G: Does CD8 depletion also increase pathogenesis in WT DENV infected animals?
9. Figure 7 B-G: Does PD-L1 antibody treatment decrease pathogenies of T209L mutant DENV infected mice?
10. In almost all graphs representative data of 2 independent experiments are shown. For the mouse experiments it would be better to combine the data form the independent experiments (n likely reflects the number of mice in these experiments). For cell culture experiments at least 3 independent experiments should be performed to enable statistical analysis.

Minor comments

1. The term "western blot" should not be capitalized.
2. Figure 2B: please show the complete lanes to demonstrate that there are no smaller complexes.
3. Figure 3C: Please change the order in the graph to WT samples on the left side as in all other graphs.
4. Page 10, lane 14: The samples were analyzed by flow cytometry not sorted through FACS.

Referee #2 (Comments on Novelty/Model System for Author):

The animal models they used are OK for DENV infection.

Referee #2 (Remarks for Author):

This is an interesting, important paper exploring the effects of glycosylation in NS1 protein of DENV in preventing the clearance mediated by CD8+T cells. However, I have several suggestions may help improve the manuscript:
1. This work is pretty much based on the evidence of antibody deletion of CD8+T cells. Cay they directly say "CD8+ T cells" in the title?
2. For Figure 5, to make the claim of the work much stronger, they may need to include a control of CD4 deletion along with CD8 deletion study. However, this may make them take extremely long to go back. Thus, they may need to make a better rationales why they selected the deletion CD8 but not CD4 in the section of "The attenuated phenotype of T209L DENV is driven by CD8+ T cells." There is a big gap from Figure 5 B to C.
3. I agree with that PD-1/L1 axis may/should play a critical role in mediating the DENV infection results (either pro-Dengue or anti-Dengue). However, I have a concern that PD-1/L1 axis may actually require to control the over-reactive inflammation during the early, acute infection stage. Also, unlike in chronic infections, T cell inhibitory molecules such as PD-1/TIM-3 may undergo functional changes in highly inflammatory disease statuses such as DENV and active Mycobacterial tuberculosis infections. Thus, for Figure 7, they may need to analyze the expressions of inflammatory cytokines to make sure that the blockade of PD-1/PD-L1 really bring down or drive up the inflammation. Also, they may need to discuss why NS1-mutant induced less expression levels of PD-1/L1 in neutrophils? Another issues is that the time course in Figure 7 B is not consistent with Figure 7 E.

***** Reviewer's comments *****

Referee #1 (Comments on Novelty/Model System for Author):

In their manuscript " Glycans on Non-Structural Protein 1 Prevent Premature T-cell 1 Mediated Dengue Virus Clearance" Idris and colleagues describe the in vivo attenuation of a DENV strain that is mutated in a specific NS1 glycosylation site. The data presented is novel and the generated hypothesis is certainly interesting to a broader audience. The strongest data presented in the manuscript are the experiments using antibody-mediated depletion of sNS1 as well as the addition sNS1 wildtype and mutant protein to demonstrate an attenuation of pathogenicity through the mutated sNS1 protein. Unfortunately, there is a major caveat in the co-infection experiments that relates to the dose of the inoculum and offers an alternative explanation. The authors have to demonstrate that this alternative explanation, i.e., that attenuation and reduced immune activation is simply due to lower inoculum size, is not true. The authors have to show that the immunological effects are indeed related to the mutated sNS1 protein and not simply reflect delayed infections kinetics due to lower inoculum of the more pathogenic wildtype strain. In addition, most data presented are from one representative experiment, it would be better to show all data.

Referee #1 (Remarks for Author):

Major Issues

1. In the co-infection experiments (Figures 4, 5, 6, and 7) the lower inoculum titer of the pathogenetic WT virus in the co-infected animals than in in the single infected animals (5 x 105 compared to 1 x 106 PFU) might explain the differences that were observed. As the WT and T209L DENV display differences in virulence (smaller plaques, lower titers in the blood and organs), the results might just reflect that the T209L mutant is less fit than the WT virus. Administering lower doses of the WT strain in co-infections might lead to differences in infection kinetics that change the immune response in mice. If there is a dominant effect of T209L mutant the same amount of WT as in the single-infected control should be administered. It would be best, however, to repeat the experiments with administered mutant sNS1 to clearly link the immune effects to the mutated sNS1 as proposed by the authors.

We have repeated the co-infection experiment using 10exp6 PFU of each virus. The results indicated that mice co-infected and mice infected with the T209L DENV2 mutant survived throughout, while mice infected with WT DENV2 met the euthanasia criteria by day 6 and 7 dpi (see below). Hence, these data confirmed that the attenuated phenotype of T209L DENV2 mutant is dominant over the virulent WT phenotype. This additional dataset has been added under Supplemental material (Fig. S2) in the revised manuscript.

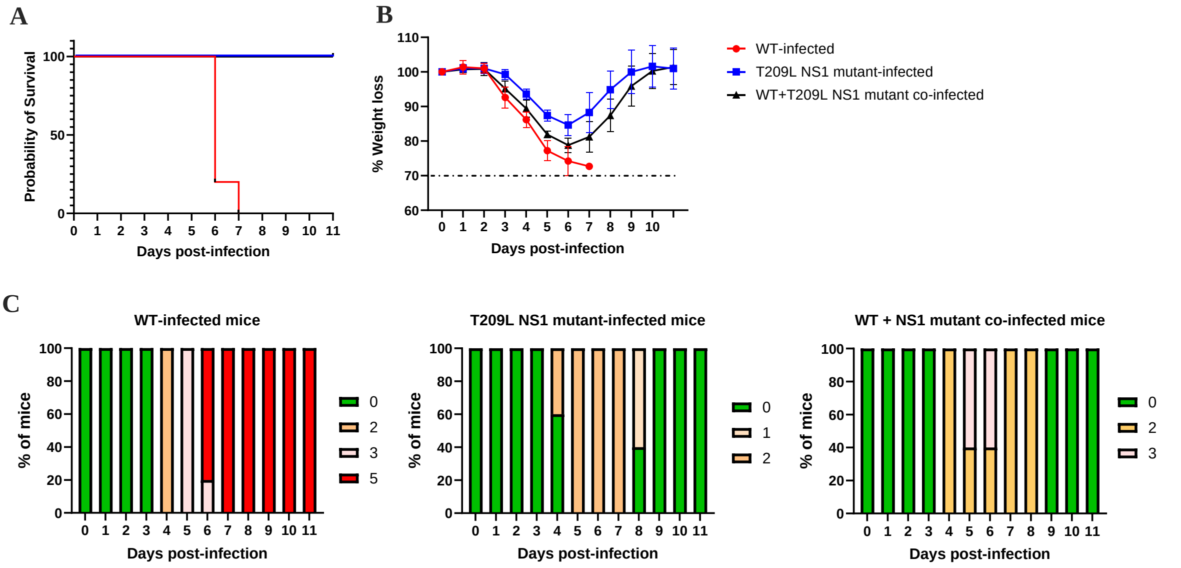

Figure. Co-infection experiment. IFNAR$^{-/-}$ mice were sc. infected with either WT or T209L mutant (10^6 PFU per mouse) or were co-infected with WT and T209L NS1 mutant viruses ($1x10^6$ PFU of each virus). (A) Survival rate (n=5), (B) Body weight profile (n=5). (C) Clinical scores as described in the legend of Fig. 1 (n=5). 0 – healthy, 1 – ruffled fur, 2 – hunched back, 3 – lethargy, 4 – limb paralysis, 5 – mice displaying 30% weight loss (euthanasia).

2. The plaque assays in the Supplement indicates different fitness levels of WT and T209L DENV strains even though the titers in vitro are similar. In addition, in Vero cells the infection kinetics seem to be a bit slower for the mutant. Even small changes in infection kinetics in vitro might account for the differences in infection kinetics and pathogenesis in vivo. Triplicate datapoints in cell culture have to be replaced by independent experiments to allow for definite conclusions of in vitro fitness (see also below).

In vitro infection experiments in cell lines allow study the ability of a virus to complete its infection cycle, from entry to exit in a specific host cell. It does not allow the evaluation of virus fitness in more complex environments such as the mammalian host where different cell types and a functional immune system are present. For this reason, it has been well accepted that *in vitro* viral fitness does not predict *in vivo* viral fitness.

Our *in vitro* results indicated that there were no significant differences between N130Q DENV, T209L DENV and WT DENV in the various cell lines tested, indicating that the lack of N130- or N207-linked glycans on NS1 did not significantly impair the ability of DENV to enter and replicate inside these cells. A biological repeat is shown below.

We respectfully disagree with the suggestion to combine datapoints from several independent experiments (biological replicates) on the same graphs. We indeed believe that showing a representative experiment (in which the statistics are conducted based on datapoints from the technical triplicates) of at least two independent experiments is the most accurate way to feature our results. Combining the absolute values obtained from several biological replicates would inevitably lead to greater intra-group variations, which may be inconclusive. Only when relative data are plotted (in reference to an internal control), data points from several independent experiments can be plotted together.

Instructions from EMBO website are as follows: “For each experiment, the number of both technical and biological replicates should be clearly stated. Biological replicates are derived from independent experiments using separately obtained biological samples, while technical replicates are created by repeated measurements on the same biological sample. In general, technical replicates should be averaged before any statistical inference tests are performed” (https://www.embopress.org/page/journal/14602075/authorguide#figureformat)

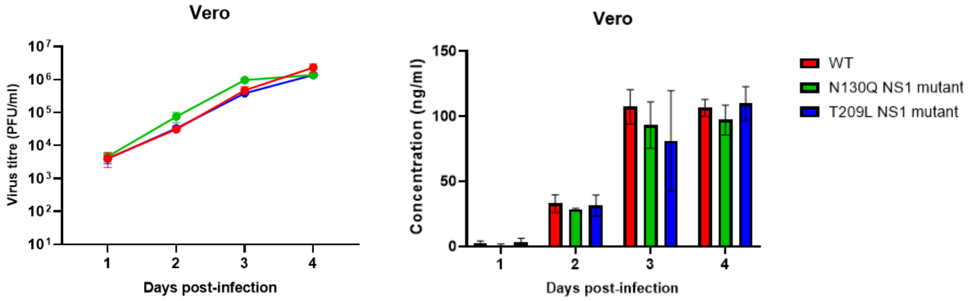

Biological repeat. Viral titers in Vero cells infected with WT, N130Q or T209L DENV2.

3. Page 7, lane 26: lower titers are first seen at day 3 (not significant) and are significantly lower at day 4, 5, and 6. This is important as the follow up experiments were performed at day 3 when small changes in titers in blood and significant changes in the organs are seen.

Viremia titers at day 3 pi. are not statistically different between WT- and T209L-infected mice. The data from the biological repeat is shown below. Hence, we concluded that at day 3 p.i. viremia titers are comparable in both infected groups.

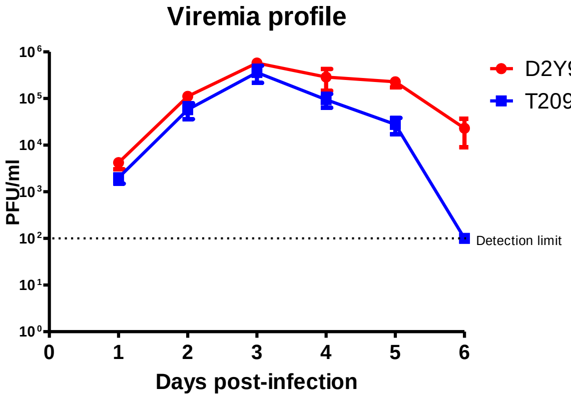

Biological repeat. Viremia profile in IFNAR$^{-/-}$ mice infected with 10^6 PFU of WT or T209L DENV2.

4. The N130Q DENV mutant is attenuated as well (viral titers in organs), therefore the statement that lower sNS1 levels do not contribute to attenuation is misleading.

We agree that significantly lower viral titers were indeed measured in the kidneys, spleen and lymph nodes at day 4 p.i. in mice infected with N130Q mutant compared to WT-infected mice. We have included this observation in the revised manuscript (page 7 line 22-23). In contrast, the sNS1 levels were significantly lower compared to WT at all the time points tested. Nevertheless, the disease severity of N130Q-infected mice was comparable to WT-infected mice based on clinical scores and body weight loss profile. Hence, we can conclude

that lower organ viral titers at day 4 p.i. and lower sNS1 levels in N130Q-infected mice did not influence disease severity. Since N130Q- and T209L-infected groups had comparable sNS1 levels, the conclusion that lower sNS1 levels in circulation do not explain the attenuated phenotype of T209L DENV mutant remains valid.

5. Figure 4: Detailed characterization of the sNS1 protein that was administered in the mouse experiments is missing, or is that the same preparation that was used in Figure 2?

Yes, the purified NS1 preparations used in Figure 4 experiment are the same ones that were used for Figure 2 experiment.

6. Figure 2C: The data presented indicate that there are less lipids bound to the T209L sNS1 even though the authors state otherwise in their discussion. In many instances the difference in lipid species levels is not statistically significant, but there is the tendency of lower lipid levels bound to the mutated sNS1. Analysis at lipid class level could reveal if there are relevant changes. In addition, the number of independent experiments is missing, if the data represents replicates and not independent experiments the statistical analysis is problematic.

We acknowledge that there is indeed a trend in less lipid content for the T209L NS1. Since we have purchased the purified NS1 preparations from a commercial vendor (custom-made), here the data are representative of technical replicates generated from the same NS1 preparations. We have clarified this in the figure legend. We have also acknowledged the trend in lower lipid content in T209L NS1 compared to WT NS1 in the revised manuscript (page 8 line 21-22).

7. Page 9, lane 23 and Figure 4C: Contrary to what is stated, viremia titers are not lowered by administration of T209L mutant sNS1 to WT infected animals.

We acknowledge that the viremia titers in WT-infected mice administered with purified T209L NS1 were not statistically lower compared to WT-infected mice, although 3 animals out of 5 had undetectable viremia at day 6 p.i. This difference did not reach statistical significance compared to the WT. We believe that these lower viremia titers are responsible for the improved clinical outcome observed in these mice. Nevertheless, we agree that exogenous administration of T209L NS1 in WT-infected mice did not fully recapitulate the attenuated phenotype of T209L DENV mutant. Based on this observation, we propose that the attenuating effect of T209L NS1 may rely on both secreted and intracellular NS1 molecules. This hypothesis is supported by the observation that the attenuated phenotype is fully recapitulated in co-infection experiments. We have included this interpretation in the revised manuscript (page 9, line 26).

8. Figure 5 C-G: Does CD8 depletion also increase pathogenesis in WT DENV infected animals?

Yes, CD8+ T-cell depletion worsened disease outcome in WT DENV-infected IFNAR$^{-/-}$ mice, which correlated with increased viremia titers at day 6 pi. (see figure below). This is consistent with previous literature (Yauch et al., 2009. doi:10.4049/jimmunol.0801974). We have revisited the interpretation of the data as follows: "Since CD8^{+} T cells have been shown to play an important role in viral clearance during primary dengue (Yauch et al, 2009) while CD4^{+} T cells were dispensable (Yauch et a., 2010), a CD8^{+} T-cell depletion experiment was

conducted to further evaluate the role of this cell subset during infection with WT or T209L DENV mutant. CD8+ T-cell depleted animals displayed increased disease severity as evidenced by increased mortality rate and increased clinical scores, which correlated with increased viremia titers (Fig. 5C-G and Fig. S3). This result thus confirmed the protective role of CD8+ T cells in our symptomatic DENV2 mouse model and indicated that the attenuated phenotype of T209L DENV2 depends on CD8+ T cells." (revised manuscript, page 11 line 8-15). This additional dataset has been included in the revised manuscript under Supplemental Figure S3.

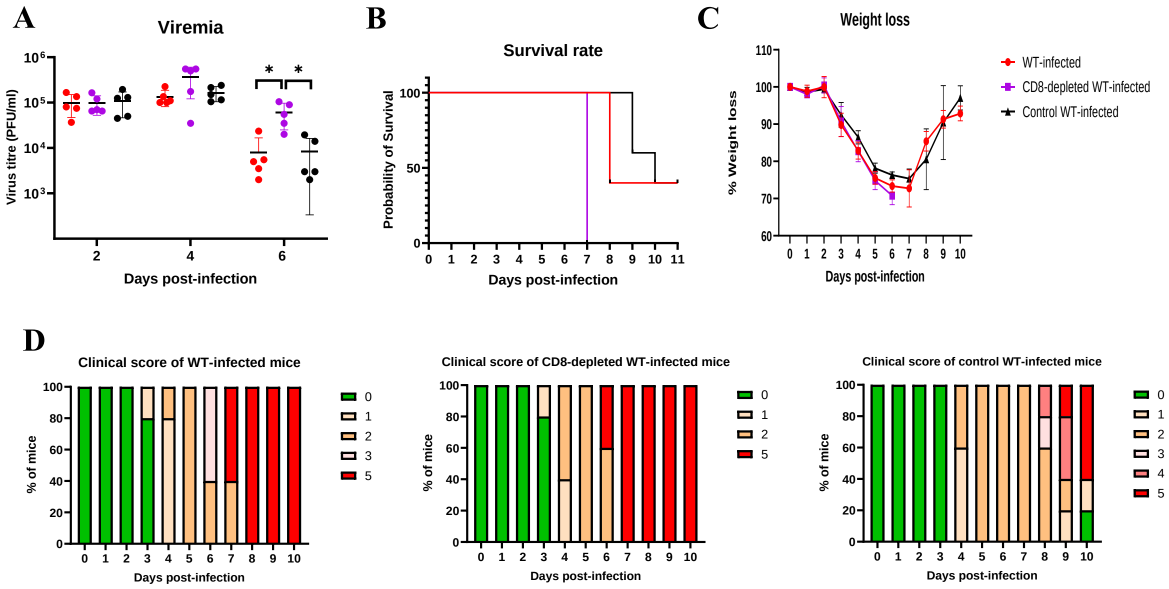

Figure. CD8+ T-cell depletion in WT DENV-infected mice. (A) Viremia titers were measured by plaque assay (n=5). (B) Survival rate (n=5). (C) Body weight profile (n=5). (D) Clinical scores as described in the legend of Fig. 1 (n = 5).

9. Figure 7 B-G: Does PD-L1 antibody treatment decrease pathogenies of T209L mutant DENV infected mice?

To explore the role of PD-1 and PD-L1, we initially performed an experiment where mice were treated either with PD-1 mAb alone or in combination with PD-L1 (combo). Results indicated that PD-1 mAb treatment significantly improved disease outcome as evidenced by 80% survival rate, milder clinical scores, improved body weight profiles and lower viremia titers at day 6 pi. compared to isotype-treated and infected only controls (Figure below). However, treatment with the combo (PD-1+PD-L1 mAbs) did not further improve the disease severity and instead worsened the disease outcome compared to PD-1 treatment alone (40% survival rate only, more severe clinical manifestations and no significant reduction in viremia titers). Previous studies have reported detrimental effects of PD-L1 blockade. Dong et al. reported that engagement of PD-L1 by an anti-PD-L1 monoclonal antibody on CD4+ T cells co-stimulated proliferation and secretion of IL-10, and subsequently led to programmed cell death, accompanied by upregulated expression of TNF-related apoptosis-inducing ligand and activation of caspase-3 (Dong et al., 2003. doi: 10.1172/JCI16015.). In cancer, some PD-L1 mAbs were reported to lose their antitumor activity by depleting PD-L1+ tumor-reactive CD8+ T cells via the p38 MAPK pathway (Liu et al., 2016 doi: 10.1038/srep36722). In bacterial infection, PD-L1 blockade inhibited the CD8+ T cell response to *Listeria monocytogenes* infection impaired bacterial clearance, while PD-1 blockade enhanced

bacterial clearance in the spleen and liver (Xu *et al.,* 2013 doi: 10.1371/journal.pone.0056539).

Hence, although we did not evaluate the effect of PD-L1 treatment alone, we believe that targeting PD-L1 in dengue may not represent a promising intervention approach.

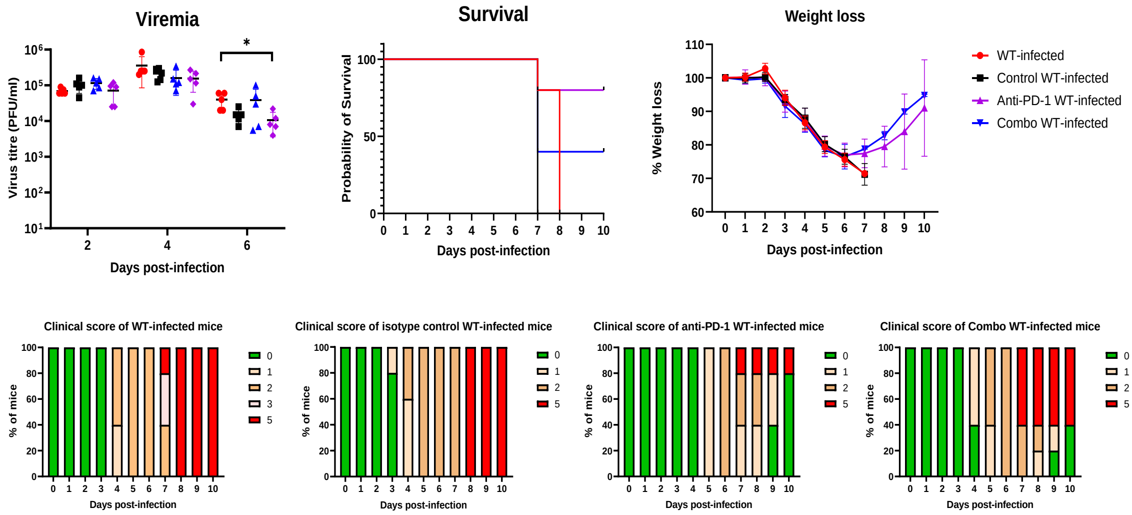

Figure. PD-L1 and PD-1 blocking in DENV-infected IFNAR$^{-/-}$ mice. 200µg of anti-PD-1 antibody or rat IgG2 isotype or a combination of anti-PD-L1 and PD-1 antibodies (combo) were administered intraperitoneally to 5-6 weeks old IFNAR mice at day 1 prior to infection, day 1, 3 and 5 pi. The mice were infected with WT at 10^6 PFU/mouse through subcutaneous route. Serum was collected through retro-orbital bleeding up to 6 days post-infection to assess the viremia. 0 – healthy, 1 – ruffled fur, 2 – hunched back, 3 – limb weakness, 4 – limb paralysis, 5 – mice displaying 30% weight loss/ succumbed to infection.

10. In almost all graphs representative data of 2 independent experiments are shown. For the mouse experiments it would be better to combine the data form the independent experiments (n likely reflects the number of mice in these experiments). For cell culture experiments at least 3 independent experiments should be performed to enable statistical analysis.

As explained above, we disagree with the suggestion to plot absolute value datapoints from several biological replicates on the same graph. In majority of scientific studies, authors present their findings from one biological replicate that is representative of at least 2 independent experiments (biological replicate). A third biological replicate is warranted when the first two repeats show discrepant trends. This is also in line with our institutional IACUC guidelines.

Minor comments

1. The term "western blot" should not be capitalized. Amended.
2. Figure 2B: please show the complete lanes to demonstrate that there are no smaller complexes. Added.
3. Figure 3C: Please change the order in the graph to WT samples on the left side as in all other graphs. Amended.
4. Page 10, lane 14: The samples were analyzed by flow cytometry not sorted through

FACS. Amended.

Referee #2 (Comments on Novelty/Model System for Author):

The animal models they used are OK for DENV infection.

Referee #2 (Remarks for Author):

This is an interesting, important paper exploring the effects of glycosylation in NS1 protein of DENV in preventing the clearance mediated by CD8+T cells. However, I have several suggestions may help improve the manuscript:

1. This work is pretty much based on the evidence of antibody deletion of CD8+T cells. Cay they directly say "CD8+ T cells" in the title?

While our data support the involvement of CD8+ T cells in the attenuated phenotype of T209L DENV mutant, we cannot rule out the role of other T cell subsets, specifically CD4+ T cells. Indeed, we observed that the number of both CD4+ and CD8+ T-cells were higher in mice infected with T209L DENV mutant compared to WT-infected mice (Fig. 5A). Furthermore, DENV-infected mice displayed higher levels of both PD-1+ CD4+ and PD-1+ CD8+ T cells (Fig. EV5). Consistently, PD-1 blockade treatment improved both CD4+ and CD8+ T cell subsets in DENV-infected mice (Fig. 7G). Hence, based on these observations, we cannot rule out a role or involvement of CD4+ T cells in the attenuated phenotype of T209L DENV. We decided to examine more closely the involvement of CD8+ T cells because this subset was previously shown to exert a protective role in primary dengue by controlling the vial titers (Yauch et al., 2009. doi:10.4049/jimmunol.0801974), while CD4+ T cells did not contribute to viral clearance (Yauch et al., 2010. doi: 10.4049/jimmunol.1001709).

Therefore, we have renamed the section title as "**The attenuated phenotype of T209L DENV involves T cells"** (revised manuscript, page 10 line 17), and we have rephrased the main conclusion of this section as follows: "Together, these observations supported that the attenuated phenotype observed in mice infected with T209L DENV mutant was driven by T cells, possibly through more effective viral clearance by CD8+ T cells." (revised manuscript, page 11 line 19).

We also believe that the main and novel finding of our work is not the involvement of CD8+ or CD4+ T cells, but more the PD-L1/PD-1 axis in dengue pathogenesis that leads to premature T-cell apoptosis. Hence, the title of our manuscript emphasizes this aspect.

2. For Figure 5, to make the claim of the work much stronger, they may need to include a control of CD4 deletion along with CD8 deletion study. However, this may make them take extremely long to go back. Thus, they may need to make a better rationales why they selected the deletion CD8 but not CD4 in the section of "The attenuated phenotype of T209L DENV is driven by CD8+ T cells." There is a big gap from Figure 5 B to C.

We agree with this Reviewer's comment and have now provided a rationale for focusing on CD8+ T cells. Page 11 line 8 of the revised manuscript: "Since $CD8^+$ T cells have been shown to play an important role in viral clearance during primary dengue (Yauch et al., 2009) while $CD4^+$ T cells were dispensable (Yauch et al., 2010), a $CD8^+$ T-cell depletion experiment was conducted to further evaluate the role of this cell subset during infection with WT or T209L DENV mutant."

We have also renamed the section: “**The attenuated phenotype of T209L DENV involves T cells** “(page 10, line 17) to account for a potential role of CD4+ T cells.

3. I agree with that PD-1/L1 axis may/should play a critical role in mediating the DENV infection results (either pro-Dengue or anti-Dengue). However, I have a concern that PD-1/L1 axis may actually require to control the over-reactive inflammation during the early, acute infection stage. Also, unlike in chronic infections, T cell inhibitory molecules such as PD-1/TIM-3 may undergo functional changes in highly inflammatory disease statuses such as DENV and active Mycobacterial tuberculosis infections. Thus, for Figure 7, they may need to analyze the expressions of inflammatory cytokines to make sure that the blockade of PD-1/PD-L1 really bring down or drive up the inflammation.

We have now measured a panel of 13 inflammatory cytokines in the serum samples of WT-infected mice treated with PD-1 mAb, isotype control or left untreated (figure below). The results indicated a transient and mild decrease in TNFα levels at day 4 p.i. in PD-1mAb-treated mice compared to the control groups (isotype-treated and untreated). No significant differences were observed between the three infected groups for the other 12 cytokines measured at this time point, and no significant differences between the three infected groups were measured at the other time points. These results indicated that PD-1 mAb treatment did not significantly alter the inflammatory cytokine profile in WT-infected mice. This is not surprising since PD-1 mAb treatment is expected to interfere with PD-L1/PD-1 interactions between innate and T-cells but is not expected to influence the pro-inflammatory cytokine production that is mainly contributed by innate immune cells. Nevertheless, PD-1 mAb treatment did lead to significant increase in T cells (CD4+ and CD8+) (Fig. 7G), which correlated with lower viremia titers at day 6 p.i. (Fig. 7F). Hence, these data indicated that the protective effect of PD-1 mAb in DENV-infected mice is not through the suppression of pro-inflammatory cytokine responses, but by blocking T-cell apoptosis.

We decided not to include this additional dataset in our revised manuscript as we think that it does not bring further insights on the protective role of NS1 T209L.

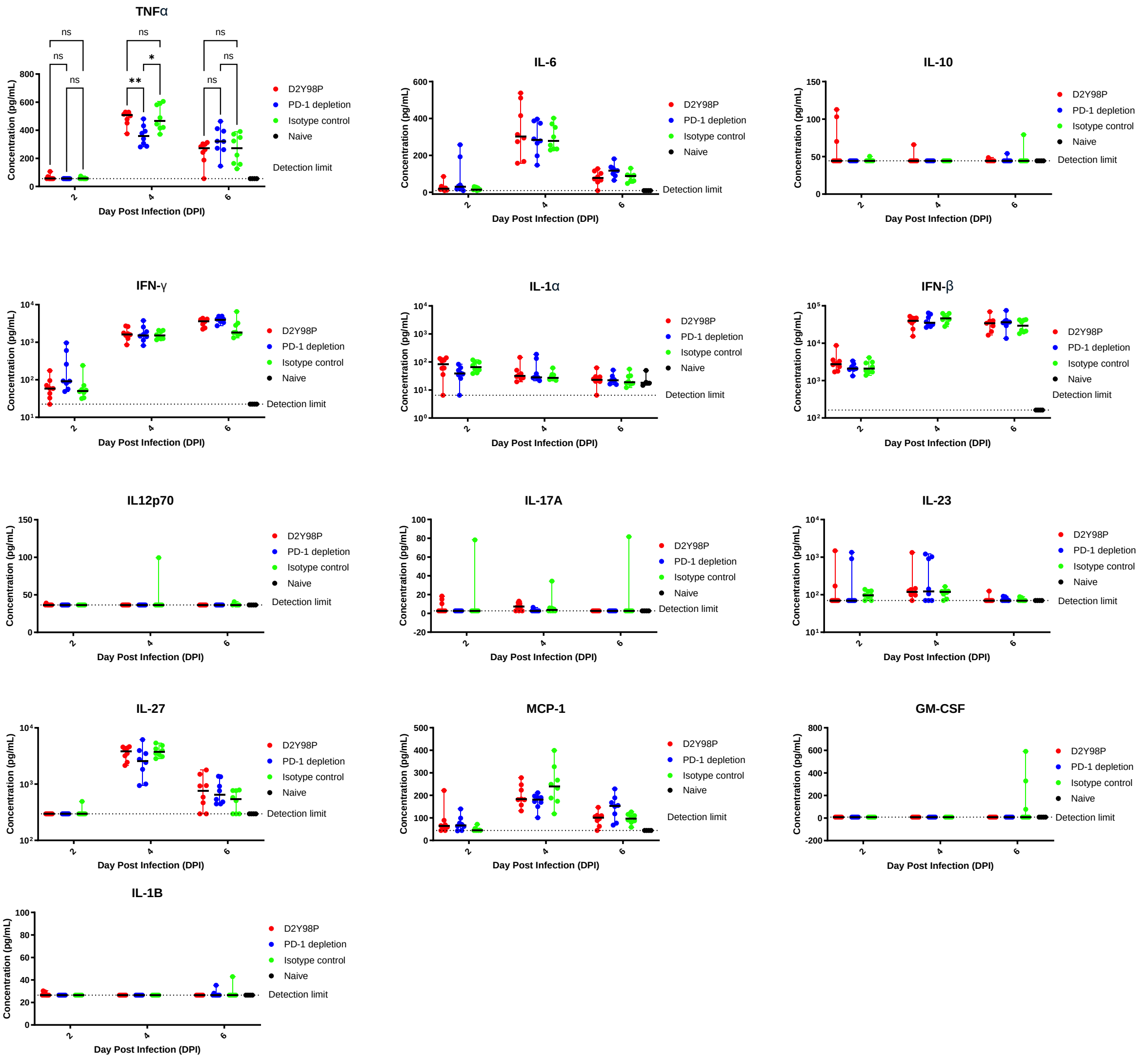

Figure. Systemic inflammatory cytokine levels in WT-infected IFNAR$^{-/-}$ mice treated with PD-1 mAb or isotype control.

Also, they may need to discuss why NS1-mutant induced less expression levels of PD-1/L1 in neutrophils?

Our mechanistic hypothesis is that T209L NS1 suppresses the production of pro-inflammatory cytokines including TNFα, IL-6 and IFNγ, which in turn limits the upregulation of PD-L1 expression not only on neutrophils but also on other innate immune cells such as DCs, monocytes, as shown in Fig. 7A and Fig. EV4. Indeed, it was shown before that PD-L1 expression depends on these pro-inflammatory cytokines (doi:10.3389/fimmu.2016.00550). This explanation can be found in our manuscript results section (page 13 line 7).

Another issues is that the time course in Figure 7 B is not consistent with Figure 7 E.

Fig. 7B has been amended to include the 10-day monitoring period.

25th Jul 2025

Dear Prof. Alonso,

Thank you for the submission of your revised manuscript to EMBO Molecular Medicine. We have now heard back from the two referees who agreed to re-evaluate your manuscript. As you will see from the reports below, while referee #2 recommends publication of the revised manuscript, referee #1 remains critical regarding the unclarity about the nature of the replicates and statistical analyses. We concluded that raised concerns are justified and should be addressed in an additional and final round of major revision. Providing information about the nature of the replicates and performing appropriate statistical tests is essential for further consideration of the manuscript. In addition, for the next submission please adhere to the journal's formatting requirements. Please check our Author Guidelines for more information
https://www.embopress.org/page/journal/17574684/authorguide#manuscriptpreparation

Please also amend following points:
1) In the main manuscript file, please do the following:
- Please address all comments suggested by our data editors listed below:
o Figure legends:
1. Please define the annotated p values ****/***/**/* as well as provide the exact p-values for the same in the legend of figure 5B, EV2 B as appropriate.
2. Please note that the exact p values are not provided in the legends of figures 4F, 5A, 6E; 7A, G.
3. Please indicate the statistical test used for data analysis in the legends of figures 4C, F; 5A, B, G; 6E, F; EV2 B, EV5.
4. Please note that information related to n is missing in the legends of figures 5A.
5. Please note that the error bars are not defined in the legends of figures 1D, F, G, H; 3B-D; 4B, C, F, H; 5A, B, E, G; 6E, F; 7A, D, F, G; EV2 A, B; EV5.
6.
Further consideration of a revision that addresses reviewer's concerns in full will entail an additional round of review. Acceptance or rejection of the manuscript will depend on the completeness of your responses included in the next, final version of the manuscript. For this reason, and to save you from any frustrations in the end, I would strongly advise against returning an incomplete revision.

We would welcome the submission of a revised version within three months for further consideration. Please let us know if you require longer to complete the revision.

I look forward to receiving your revised manuscript.

Yours sincerely,

Zeljko Durdevic

Zeljko Durdevic
Senior Editor
EMBO Molecular Medicine

*****

When submitting your revised manuscript, please carefully review the instructions that follow below. We perform an initial quality control of all revised manuscripts before re-review; failure to include requested items will delay the evaluation of your revision.

We require:

1) A .docx formatted version of the manuscript text (including legends for main figures, EV figures and tables). Please make sure that the changes are highlighted to be clearly visible.

2) Individual production quality figure files as .eps, .tif, .jpg (one file per figure). For guidance, download the 'Figure Guide PDF': (https://www.embopress.org/page/journal/17574684/authorguide#figureformat).

3) A .docx formatted letter INCLUDING the reviewers' reports and your detailed point-by-point responses to their comments. As part of the EMBO Press transparent editorial process, the point-by-point response is part of the Review Process File (RPF), which will be published alongside your paper.

4) A complete author checklist, which you can download from our author guidelines (https://www.embopress.org/page/journal/17574684/authorguide#submissionofrevisions). Please insert information in the checklist that is also reflected in the manuscript. The completed author checklist will also be part of the RPF.

5) Please note that all corresponding authors are required to supply an ORCID ID for their name upon submission of a revised manuscript.

6) It is mandatory to include a 'Data Availability' section after the Materials and Methods. Before submitting your revision, primary datasets produced in this study need to be deposited in an appropriate public database, and the accession numbers and database listed under 'Data Availability'. Please remember to provide a reviewer password if the datasets are not yet public (see https://www.embopress.org/page/journal/17574684/authorguide#dataavailability).

In case you have no data that requires deposition in a public database, please state so in this section. Note that the Data Availability Section is restricted to new primary data that are part of this study.

7) For data quantification: please specify the name of the statistical test used to generate error bars and P values, the number (n) of independent experiments (specify technical or biological replicates) underlying each data point and the test used to calculate p-values in each figure legend. The figure legends should contain a basic description of n, P and the test applied. Graphs must include a description of the bars and the error bars (s.d., s.e.m.). See also 'Figure Legend' guidelines: https://www.embopress.org/page/journal/17574684/authorguide#figureformat

8) At EMBO Press we ask authors to provide source data for the main manuscript figures. You will receive a separate email with instructions for providing source data with your revised manuscript, including how to upload and organize the files.

9) Our journal encourages inclusion of *data citations in the reference list* to directly cite datasets that were re-used and obtained from public databases. Data citations in the article text are distinct from normal bibliographical citations and should directly link to the database records from which the data can be accessed. In the main text, data citations are formatted as follows: "Data ref: Smith et al, 2001" or "Data ref: NCBI Sequence Read Archive PRJNA342805, 2017". In the Reference list, data citations must be labeled with "[DATASET]". A data reference must provide the database name, accession number/identifiers and a resolvable link to the landing page from which the data can be accessed at the end of the reference. Further instructions are available at .

10) We replaced Supplementary Information with Expanded View (EV) Figures and Tables that are collapsible/expandable online. A maximum of 5 EV Figures can be typeset. EV Figures should be cited as 'Figure EV1, Figure EV2" etc... in the text and their respective legends should be included in the main text after the legends of regular figures.

- For the figures that you do NOT wish to display as Expanded View figures, they should be bundled together with their legends in a single PDF file called *Appendix*, which should start with a short Table of Content. Appendix figures should be referred to in the main text as: "Appendix Figure S1, Appendix Figure S2" etc.

- Additional Tables/Datasets should be labeled and referred to as Table EV1, Dataset EV1, etc. Legends have to be provided in a separate tab in case of .xls files. Alternatively, the legend can be supplied as a separate text file (README) and zipped together with the Table/Dataset file.

See detailed instructions here:

.

11) The paper explained: EMBO Molecular Medicine articles are accompanied by a summary of the articles to emphasize the major findings in the paper and their medical implications for the non-specialist reader. Please provide a draft summary of your article highlighting

- the medical issue you are addressing,

- the results obtained and

- their clinical impact.

This may be edited to ensure that readers understand the significance and context of the research. Please refer to any of our published articles for an example.

12) Author contributions: You will be asked to provide CRediT (Contributor Role Taxonomy) terms in the submission system. These replace a narrative author contribution section in the manuscript.

13) A Conflict of Interest statement should be provided in the main text.

14) Every published paper now includes a 'Synopsis' to further enhance discoverability. Synopses are displayed on the journal webpage and are freely accessible to all readers. They include a short stand first (maximum of 300 characters, including space) as well as 2-5 one-sentences bullet points that summarizes the paper. Please write the bullet points to summarize the key NEW findings. They should be designed to be complementary to the abstract - i.e. not repeat the same text. We encourage inclusion of key acronyms and quantitative information (maximum of 30 words / bullet point). Please use the passive voice. Please attach these in a separate file or send them by email, we will incorporate them accordingly.

Please also suggest a striking image or visual abstract to illustrate your article as a PNG file 550 px wide x 300-600 px high.

15) Include a Reagents and Tools Table as part of the Methods section, which can be downloaded from our author guidelines (https://www.embopress.org/page/journal/17574684/authorguide#structuredmethods)

EMBO Molecular Medicine has a "scooping protection" policy, whereby similar findings that are published by others during review or revision are not a criterion for rejection. Should you decide to submit a revised version, I do ask that you get in touch after three months if you have not completed it, to update us on the status.

***** Reviewer's comments *****

Referee #1 (Remarks for Author):

In my initial review, I requested that the authors perform statistically valid analyses based on independent biological experiments, rather than showing technical replicates. While the authors state that they repeated the experiments with similar results, the journal's editorial policies are explicit in distinguishing between technical and biological replicates. As per the guidelines:
"For each experiment, the number of both technical and biological replicates should be clearly stated. Biological replicates are derived from independent experiments using separately obtained biological samples, while technical replicates are created by repeated measurements on the same biological sample. In general, technical replicates should be averaged before any statistical inference tests are performed."
Furthermore:
"The description of all reported data that includes statistical testing must state the name of the statistical test used to generate error bars and P values, the number (n) of independent experiments underlying each data point (not replicate measures of one sample), and the actual P value for each test (not merely 'significant' or 'P < 0.05')."
This issue was discussed with the editor, and I emphasize again that performing statistical tests on technical replicates does not meet the standards outlined by the journal. It is essential that the authors conduct independent biological experiments and present statistical analyses based on those data. This was one of my principal concerns in my initial review.
I acknowledge that all other issues raised previously have been adequately addressed. However, this core issue regarding the nature of the replicates and the validity of the statistical analysis remains unresolved and must be corrected to comply with the journal's editorial policies.

Referee #2 (Comments on Novelty/Model System for Author):

The animal model and in vitro model for DENV infections are OK.

Referee #2 (Remarks for Author):

I have no more comment as they have addressed most of my previous comments.

***** Reviewer's comments *****

Referee #1 (Remarks for Author):

In my initial review, I requested that the authors perform statistically valid analyses based on independent biological experiments, rather than showing technical replicates. While the authors state that they repeated the experiments with similar results, the journal's editorial policies are explicit in distinguishing between technical and biological replicates. As per the guidelines:
"For each experiment, the number of both technical and biological replicates should be clearly stated. Biological replicates are derived from independent experiments using separately obtained biological samples, while technical replicates are created by repeated measurements on the same biological sample. In general, technical replicates should be averaged before any statistical inference tests are performed."
Furthermore:
"The description of all reported data that includes statistical testing must state the name of the statistical test used to generate error bars and P values, the number (n) of independent experiments underlying each data point (not replicate measures of one sample), and the actual P value for each test (not merely 'significant' or 'P < 0.05')."
This issue was discussed with the editor, and I emphasize again that performing statistical tests on technical replicates does not meet the standards outlined by the journal. It is essential that the authors conduct independent biological experiments and present statistical analyses based on those data. This was one of my principal concerns in my initial review.
I acknowledge that all other issues raised previously have been adequately addressed. However, this core issue regarding the nature of the replicates and the validity of the statistical analysis remains unresolved and must be corrected to comply with the journal's editorial policies.

We have consolidated data points from multiple independent experiments where suitable and conducted the necessary statistical analyses, displaying the exact p values on the graphs. Please note that, for some graphs, p values are shown as p<0.0001 due to limitations of the software (PRISM). In each figure legend, we have clearly specified the number of independent experiments, technical replicates, or mice used, as well as the statistical test applied.

26th Aug 2025

Dear Prof. Alonso,

Thank you for the submission of your revised manuscript to EMBO Molecular Medicine. I am pleased to inform you that we will be able to accept your manuscript pending the following final amendments:

1) Figures: Please upload main figures and EV figures as individual high-resolution files. Please check "Author Guidelines" for more information:
https://www.embopress.org/page/journal/17574684/authorguide#figureformat
https://www.embopress.org/page/journal/17574684/authorguide#expandedview
2) In the main manuscript file, please do the following:
- Please address all comments suggested by our data editors listed below:
o Figure legends:
1. Please define the annotated p values ****/***/**/* as well as provide the exact p-values for the same in the legend of figure 5B, EV2 B as appropriate.
2. Please note that the exact p values are not provided in the legends of figures 4F, 5A, 6E; 7A, G.
3. Please indicate the statistical test used for data analysis in the legends of figures 4C, F; 5A, B, G; 6E, F; EV2 B, EV5.
4. Please note that information related to n is missing in the legends of figures 5A.
5. Please note that the error bars are not defined in the legends of figures 1D, F, G, H; 3B-D; 4B, C, F, H; 5A, B, E, G; 6E, F; 7A, D, F, G; EV2 A, B; EV5.
- Add callouts for the Figure 3A-B, 4I and 7B and make sure that the figures and panels are called out in a sequential order. Currently Figure 3C is called out before Figure 3B. Please correct.
- Please remove Reagents and Tools Table form the manuscript file and upload it as a separate file. More information on how to adhere to this format as well as downloadable templates (.docx) for the Reagents and Tools Table can be found in our author guidelines:
https://www.embopress.org/page/journal/17574684/authorguide#structuredmethods
An example of a paper with Structured Methods can be found here:
https://www.embopress.org/doi/full/10.1038/s44320-024-00037-6#sec-4
- Author contributions: Please remove it from the manuscript and specify author contributions in our submission system. CRediT has replaced the traditional author contributions section because it offers a systematic machine-readable author contributions format that allows for more effective research assessment. You are encouraged to use the free text boxes beneath each contributing author's name to add specific details on the author's contribution. More information is available in our guide to authors:
https://www.embopress.org/page/journal/17574684/authorguide#authorshipguidelines
- In Methods, statistical paragraph should reflect all information that you have filled in the Authors Checklist, especially regarding randomization, blinding, replication etc.
- Indicate in legends exact n and exact p values, not a range, along with the statistical test used. To keep the figures "clear" some authors found providing an Appendix table Sx with all exact p-values preferable. You are welcome to do this if you want to.
- Move the data availability statement to the end of the Methods section. Please use the following format to report the accession number of your data:

The datasets produced in this study are available in the following databases:
[data type]: [full name of the resource] [accession number/identifier] ([doi or URL or identifiers.org/DATABASE:ACCESSION])

Please check "Author Guidelines" for more information.
https://www.embopress.org/page/journal/17574684/authorguide#availabilityofpublishedmaterial
3) Appendix: Please rename Suppl. Materials to "Appendix" and add a table of content with page numbers on the title page. Correct the nomenclature to "Appendix Table S1" etc., and "Appendix Figure S1" etc. and updated their callouts in the main manuscript file.
4) Source data: Please upload one zipped file per figure.
5) Synopsis: Every published paper now includes a 'Synopsis' to further enhance discoverability. Synopses are displayed on the journal webpage and are freely accessible to all readers. They include separate synopsis image and synopsis text.
- Synopsis image: Please provide a visual abstract as a high-resolution jpeg file 550 px-wide x (300-600)-px high to illustrate your article.
- Synopsis text: Please provide a short standfirst (maximum of 300 characters, including space) as well as 2-5 one sentence bullet points that summarise the paper as a .doc file. Please write the bullet points to summarise the key NEW findings. They should be designed to be complementary to the abstract - i.e. not repeat the same text. We encourage inclusion of key acronyms and quantitative information (maximum of 30 words / bullet point). Please use the passive voice.
- Please check your synopsis text and image before submission with your revised manuscript. Please be aware that in the proof stage minor corrections only are allowed (e.g., typos).
6) As part of the EMBO Publications transparent editorial process initiative (see our Editorial at

http://embomolmed.embopress.org/content/2/9/329), EMBO Molecular Medicine will publish online a Review Process File (RPF) to accompany accepted manuscripts. This file will be published in conjunction with your paper and will include the anonymous referee reports, your point-by-point response and all pertinent correspondence relating to the manuscript. Let us know whether you agree with the publication of the RPF and as here, if you want to remove or not any figures from it prior to publication. Please note that the Authors checklist will be published at the end of the RPF.
7) Please provide a point-by-point letter INCLUDING my comments as well as the reviewer's reports and your detailed responses (as Word file).

I look forward to reading a new revised version of your manuscript as soon as possible.

Yours sincerely,

Zeljko Durdevic

Zeljko Durdevic
Senior Editor
EMBO Molecular Medicine

*** Instructions to submit your revised manuscript ***

*** PLEASE NOTE *** As part of the EMBO Publications transparent editorial process initiative (see our Editorial at https://www.embopress.org/doi/pdf/10.1002/emmm.201000094), EMBO Molecular Medicine will publish online a Review Process File to accompany accepted manuscripts.

In the event of acceptance, this file will be published in conjunction with your paper and will include the anonymous referee reports, your point-by-point response and all pertinent correspondence relating to the manuscript. If you do NOT want this file to be published, please inform the editorial office at contact@embomolmed.org.

When submitting your revised manuscript, please include:

1) a .docx formatted version of the manuscript text (including Figure legends and tables)

2) Separate figure files*

3) supplemental information as Expanded View and/or Appendix. Please carefully check the authors guidelines for formatting Expanded view and Appendix figures and tables at
https://www.embopress.org/page/journal/17574684/authorguide#expandedview

4) a letter INCLUDING the reviewer's reports and your detailed responses to their comments (as Word file).

5) The paper explained: EMBO Molecular Medicine articles are accompanied by a summary of the articles to emphasize the major findings in the paper and their medical implications for the non-specialist reader. Please provide a draft summary of your article highlighting
- the medical issue you are addressing,
- the results obtained and
- their clinical impact.
This may be edited to ensure that readers understand the significance and context of the research.
Please refer to any of our published articles for an example.

6) Author contributions: the contribution of every author must be detailed in a separate section.

7) EMBO Molecular Medicine now requires a complete author checklist (https://www.embopress.org/page/journal/17574684/authorguide) to be submitted with all revised manuscripts. Please use the

checklist as guideline for the sort of information we need WITHIN the manuscript. The checklist should only be filled with page numbers were the information can be found. This is particularly important for animal reporting, antibody dilutions (missing) and exact values and n that should be indicted instead of a range.

8) Every published paper now includes a 'Synopsis' to further enhance discoverability. Synopses are displayed on the journal webpage and are freely accessible to all readers. They include a short stand first (maximum of 300 characters, including space) as well as 2-5 one sentence bullet points that summarise the paper. Please write the bullet points to summarise the key NEW findings. They should be designed to be complementary to the abstract - i.e. not repeat the same text. We encourage inclusion of key acronyms and quantitative information (maximum of 30 words / bullet point). Please use the passive voice. Please attach these in a separate file or send them by email, we will incorporate them accordingly.

You are also welcome to suggest a striking image or visual abstract to illustrate your article. If you do please provide a jpeg file 550 px-wide x 300-600px high.

9) A Conflict of Interest statement should be provided in the main text

10) Please note that we now mandate that all corresponding authors list an ORCID digital identifier. This takes <90 seconds to complete. We encourage all authors to supply an ORCID identifier, which will be linked to their name for unambiguous name identification.

Currently, our records indicate that the ORCID for your account is 0000-0001-7044-414X.

Please click the link below to modify this ORCID:
Link Not Available

11) Include a Reagents and Tools Table as part of the Methods section, which can be downloaded from our author guidelines (https://www.embopress.org/page/journal/17574684/authorguide#structuredmethods)

*Additional important information regarding Figures

Each figure should be given in a separate file and should have the following resolution:
Graphs 800-1,200 DPI
Photos 400-800 DPI
Colour (only CMYK) 300-400 DPI"

Figures are not edited by the production team. All lettering should be the same size and style; figure panels should be indicated by capital letters (A, B, C etc). Gridlines are not allowed except for log plots. Figures should be numbered in the order of their appearance in the text with Arabic numerals. Each Figure must have a separate legend and a caption is needed for each panel.

*Additional important information regarding figures and illustrations can be found at https://bit.ly/EMBOPressFigurePreparationGuideline. See also figure legend preparation guidelines: https://www.embopress.org/page/journal/17574684/authorguide#figureformat

***** Reviewer's comments *****

Referee #1 (Remarks for Author):

I have no further comments, the authors addressed all concerns.

The authors addressed the remaining editorial issues.

3rd Sep 2025

Dear Prof. Alonso,

We are pleased to inform you that your manuscript is accepted for publication and is now being sent to our publisher to be included in the next available issue of EMBO Molecular Medicine.

Your manuscript will be processed for publication by EMBO Press. It will be copy edited and you will receive page proofs prior to publication. Please note that you will be contacted by Springer Nature Author Services to complete licensing and payment information.

You may qualify for financial assistance for your publication charges - either via a Springer Nature fully open access agreement or an EMBO initiative. Check your eligibility: https://www.embopress.org/page/journal/17574684/authorguide#chargesguide

Should you be planning a Press Release on your article, please get in contact with embo_production@springernature.com as early as possible in order to coordinate publication and release dates.

If you have any questions, please do not hesitate to contact the Editorial Office. Thank you for your contribution to EMBO Molecular Medicine.

Yours sincerely,
Zeljko Durdevic

Zeljko Durdevic
Editor
EMBO Molecular Medicine

>>> Please note that it is EMBO Molecular Medicine policy for the transcript of the editorial process (containing referee reports and your response letter) to be published as an online supplement to each paper. If you do NOT want this, you will need to inform the Editorial Office via email immediately. More information is available here: https://www.embopress.org/transparent-process#Review_Process

EMBO Press Author Checklist

Corresponding Author Name: Sylvie Alonso	
Journal Submitted to: EMBO Molecular Medicine	
Manuscript Number: EMM-2025-21543	

USEFUL LINKS FOR COMPLETING THIS FORM
The EMBO Journal - Author Guidelines
EMBO Reports - Author Guidelines
Molecular Systems Biology - Author Guidelines
EMBO Molecular Medicine - Author Guidelines

Reporting Checklist for Life Science Articles (updated January
This checklist is adapted from Materials Design Analysis Reporting (MDAR) Checklist for Authors. MDAR establishes a minimum set of requirements in transparent reporting in the life sciences (see Statement of Task: 10.31222/osf.io/9sm4x). Please follow the journal's guidelines in preparing your
Please note that a copy of this checklist will be published alongside your article.

Abridged guidelines for figures
1. Data
The data shown in figures should satisfy the following conditions:
- the data were obtained and processed according to the field's best practice and are presented to reflect the results of the experiments in an accurate and unbiased manner.
- ideally, figure panels should include only measurements that are directly comparable to each other and obtained with the same assay.
- plots include clearly labeled error bars for independent experiments and sample sizes. Unless justified, error bars should not be shown for technical
- if n<5, the individual data points from each experiment should be plotted. Any statistical test employed should be justified.
- Source Data should be included to report the data underlying figures according to the guidelines set out in the authorship guidelines on Data

2. Captions
Each figure caption should contain the following information, for each panel where they are relevant:
- a specification of the experimental system investigated (eg cell line, species name).
- the assay(s) and method(s) used to carry out the reported observations and measurements.
- an explicit mention of the biological and chemical entity(ies) that are being measured.
- an explicit mention of the biological and chemical entity(ies) that are altered/varied/perturbed in a controlled manner.
- the exact sample size (n) for each experimental group/condition, given as a number, not a range;
- a description of the sample collection allowing the reader to understand whether the samples represent technical or biological replicates (including how many animals, litters, cultures, etc.).
- a statement of how many times the experiment shown was independently replicated in the laboratory.
- definitions of statistical methods and measures:
  - common tests, such as t-test (please specify whether paired vs. unpaired), simple χ2 tests, Wilcoxon and Mann-Whitney tests, can be unambiguously identified by name only, but more complex techniques should be described in the methods section;
  - are tests one-sided or two-sided?
  - are there adjustments for multiple comparisons?
  - exact statistical test results, e.g., P values = x but not P values < x;
  - definition of 'center values' as median or average;
  - definition of error bars as s.d. or s.e.m.

Please complete ALL of the questions below.
Select "Not Applicable" only when the requested information is not relevant for your study.

Materials

Newly Created Materials	Information included in the manuscript?	In which section is the information available? (Reagents and Tools Table, Materials and Methods, Figures, Data Availability Section)
New materials and reagents need to be available; do any restrictions apply?	Not Applicable	

Antibodies	Information included in the manuscript?	In which section is the information available? (Reagents and Tools Table, Materials and Methods, Figures, Data Availability Section)
For **antibodies** provide the following information:<br>- Commercial antibodies: RRID (if possible) or supplier name, catalogue number and or/clone number<br>- Non-commercial: RRID or citation	Yes	Material and Methods section.

DNA and RNA sequences	Information included in the manuscript?	In which section is the information available? (Reagents and Tools Table, Materials and Methods, Figures, Data Availability Section)
**Short novel DNA or RNA including primers, probes**: provide the sequences.	Yes	Table S2

Cell materials	Information included in the manuscript?	In which section is the information available? (Reagents and Tools Table, Materials and Methods, Figures, Data Availability Section)
**Cell lines:** Provide species information, strain. Provide accession number in repository **OR** supplier name, catalog number, clone number, and/**OR** RRID.	Yes	M&M section
**Primary cultures:** Provide species, strain, sex of origin, genetic modification status.	Not Applicable	
Report if the cell lines were recently **authenticated** (e.g., by STR profiling) and tested for mycoplasma contamination.	Yes	M&M section

Experimental animals	Information included in the manuscript?	In which section is the information available? (Reagents and Tools Table, Materials and Methods, Figures, Data Availability Section)
**Laboratory animals or Model organisms:** Provide species, strain, sex, age, genetic modification status. Provide accession number in repository **OR** supplier name, catalog number, clone number, **OR** RRID.	Yes	M&M section
**Animal observed in or captured from the field:** Provide species, sex, and age where possible.	Not Applicable	
Please detail **housing and husbandry conditions**.	Yes	M&M section

Plants and microbes	Information included in the manuscript?	In which section is the information available? (Reagents and Tools Table, Materials and Methods, Figures, Data Availability Section)
**Plants:** provide species and strain, ecotype and cultivar where relevant, unique accession number if available, and source (including location for collected wild specimens).	Not Applicable	
**Microbes:** provide species and strain, unique accession number if available, and source.	Yes	M&M section

Human research participants	Information included in the manuscript?	In which section is the information available? (Reagents and Tools Table, Materials and Methods, Figures, Data Availability Section)
If collected and within the bounds of privacy constraints report on age, sex and gender or ethnicity for all study participants.	Not Applicable	

Core facilities	Information included in the manuscript?	In which section is the information available? (Reagents and Tools Table, Materials and Methods, Figures, Data Availability Section)
If your work benefited from core facilities, was their service mentioned in the acknowledgments section?	Yes	Acknowledgements section

Design

Study protocol	Information included in the manuscript?	In which section is the information available? (Reagents and Tools Table, Materials and Methods, Figures, Data Availability Section)
If study protocol has been **pre-registered, provide DOI in the manuscript**. For clinical trials, provide the trial registration number **OR** cite DOI.	Not Applicable	
Report the **clinical trial registration number** (at ClinicalTrials.gov or equivalent), where applicable.	Not Applicable	

Laboratory protocol	Information included in the manuscript?	In which section is the information available? (Reagents and Tools Table, Materials and Methods, Figures, Data Availability Section)
Provide DOI OR other citation details if **external detailed step-by-step protocols** are available.	Not Applicable	

Experimental study design and statistics	Information included in the manuscript?	In which section is the information available? (Reagents and Tools Table, Materials and Methods, Figures, Data Availability Section)
Include a statement about **sample size** estimate even if no statistical methods were used.	Yes	M&M
Were any steps taken to minimize the effects of subjective bias when allocating animals/samples to treatment (e.g. **randomization procedure**)? If yes, have they been described?	Yes	M&M
Include a statement about **blinding** even if no blinding was done.	Yes	M&M
Describe **inclusion/exclusion criteria** if samples or animals were excluded from the analysis. Were the criteria pre-established?<br><br>If sample or data points were omitted from analysis, report if this was due to attrition or intentional exclusion and provide justification.	Not Applicable	
For every figure, are **statistical tests** justified as appropriate? Do the data meet the assumptions of the tests (e.g., normal distribution)? Describe any methods used to assess it. Is there an estimate of variation within each group of data? Is the variance similar between the groups that are being statistically compared?	Yes	M&M

Sample definition and in-laboratory replication	Information included in the manuscript?	In which section is the information available? (Reagents and Tools Table, Materials and Methods, Figures, Data Availability Section)
In the figure legends: state number of times the experiment was **replicated** in laboratory.	Yes	figure legends
In the figure legends: define whether data describe **technical or biological replicates**.	Yes	figure legends

Ethics

Ethics	Information included in the manuscript?	In which section is the information available? (Reagents and Tools Table, Materials and Methods, Figures, Data Availability Section)
Studies involving **human participants**: State details of **authority granting ethics approval** (IRB or equivalent committee(s), provide reference number for approval.	Not Applicable	
Studies involving **human participants**: Include a statement confirming that **informed consent** was obtained from all subjects and that the experiments conformed to the principles set out in the WMA Declaration of Helsinki and the Department of Health and Human Services Belmont Report.	Not Applicable	
Studies involving **human participants: For** publication of **patient photos**, include a statement confirming that consent to publish was obtained.	Not Applicable	
Studies involving experimental **animals**: State details of **authority granting ethics approval** (IRB or equivalent committee(s), provide reference number for approval. Include a statement of compliance with ethical regulations.	Yes	M&M
Studies involving **specimen and field samples:** State if relevant **permits** obtained, provide details of authority approving study; if none were required, explain why.	Not Applicable	

Dual Use Research of Concern (DURC)	Information included in the manuscript?	In which section is the information available? (Reagents and Tools Table, Materials and Methods, Figures, Data Availability Section)
Could your study fall under dual use research restrictions? Please check biosecurity documents and list of **select agents and toxins** (CDC): https://www.selectagents.gov/sat/list.htm	Not Applicable	
If you used a select agent, is the security level of the lab appropriate and reported in the manuscript?	Yes	M&M
If a study is subject to dual use research of concern regulations, is the name of the **authority granting approval and reference number** for the regulatory approval provided in the manuscript?	Not Applicable	

Reporting

The MDAR framework recommends adoption of discipline-specific guidelines, established and endorsed through community initiatives. Journals have their own policy about requiring specific guidelines and recommendations to complement MDAR.

Adherence to community standards	Information included in the manuscript?	In which section is the information available? (Reagents and Tools Table, Materials and Methods, Figures, Data Availability Section)
State if relevant guidelines or checklists (e.g., **ICMJE, MIBBI, ARRIVE, PRISMA**) have been followed or provided.	Not Applicable	
For **tumor marker prognostic studies,** we recommend that you follow the **REMARK** reporting guidelines (see link list at top right). See author guidelines, under 'Reporting Guidelines'. Please confirm you have followed these guidelines.	Not Applicable	
For **phase II and III randomized controlled trials**, please refer to the **CONSORT** flow diagram (see link list at top right) and submit the CONSORT checklist (see link list at top right) with your submission. See author guidelines, under 'Reporting Guidelines'. Please confirm you have submitted this list.	Not Applicable	

Data Availability

Data availability	Information included in the manuscript?	In which section is the information available? (Reagents and Tools Table, Materials and Methods, Figures, Data Availability Section)
Have **primary datasets** been deposited according to the journal's guidelines (see 'Data Deposition' section) and the respective accession numbers provided in the Data Availability Section?	Yes	at the end of the manuscript
Were **human clinical and genomic datasets** deposited in a public access-controlled repository in accordance to ethical obligations to the patients and to the applicable consent agreement?	Not Applicable	
Are **computational models** that are central and integral to a study available without restrictions in a machine-readable form? Were the relevant accession numbers or links provided?	Not Applicable	
If publicly available data were reused, provide the respective **data citations in the reference list**.	Not Applicable	
